# TRIM18 is a critical regulator of viral myocarditis and organ inflammation

**DOI:** 10.1186/s12929-022-00840-z

**Published:** 2022-07-31

**Authors:** Mingli Fang, Ao Zhang, Yong Du, Wenting Lu, Junying Wang, Laurie J. Minze, Timothy C. Cox, Xian Chang Li, Junji Xing, Zhiqiang Zhang

**Affiliations:** 1 Department of Surgery and Immunobiology and Transplant Science Center, Houston Methodist Research Institute, Houston Methodist Hospital, Houston, TX 77030 USA; 2 Department of Molecular Biology, College of Basic Medical Sciences, Jilin University, Changchun, 130021 China; 3 Department of Laboratory Medicine, State Key Laboratory of Oncology in South China, Collaborative Innovation Center for Cancer Medicine, Sun Yat-Sen University Cancer Center, Guangzhou, 510060 China; 4 Department of Oral & Craniofacial Sciences, School of Dentistry & Department of Pediatrics, School of Medicine, University of Missouri-Kansas City, Kansas City, MO 64108 USA; 5 Department of Surgery, Weill Cornell Medical College, Cornell University, New York, NY 10065 USA

**Keywords:** Innate immunity, RNA virus, DNA virus, Myocarditis, Inflammation, Type I IFN, TBK1, MAVS, STING, Ubiquitination

## Abstract

**Background:**

Infections by viruses including severe acute respiratory syndrome coronavirus 2 could cause organ inflammations such as myocarditis, pneumonia and encephalitis. Innate immunity to viral nucleic acids mediates antiviral immunity as well as inflammatory organ injury. However, the innate immune mechanisms that control viral induced organ inflammations are unclear.

**Methods:**

To understand the role of the E3 ligase TRIM18 in controlling viral myocarditis and organ inflammation, wild-type and Trim18 knockout mice were infected with coxsackievirus B3 for inducing viral myocarditis, influenza A virus PR8 strain and human adenovirus for inducing viral pneumonia, and herpes simplex virus type I for inducing herpes simplex encephalitis. Mice survivals were monitored, and heart, lung and brain were harvested for histology and immunohistochemistry analysis. Real-time PCR, co-immunoprecipitation, immunoblot, enzyme-linked immunosorbent assay, luciferase assay, flow cytometry, over-expression and knockdown techniques were used to understand the molecular mechanisms of TRIM18 in regulating type I interferon (IFN) production after virus infection in this study.

**Results:**

We find that knockdown or deletion of TRIM18 in human or mouse macrophages enhances production of type I IFN in response to double strand (ds) RNA and dsDNA or RNA and DNA virus infection. Importantly, deletion of TRIM18 protects mice from viral myocarditis, viral pneumonia, and herpes simplex encephalitis due to enhanced type I IFN production in vivo. Mechanistically, we show that TRIM18 recruits protein phosphatase 1A (PPM1A) to dephosphorylate TANK binding kinase 1 (TBK1), which inactivates TBK1 to block TBK1 from interacting with its upstream adaptors, mitochondrial antiviral signaling (MAVS) and stimulator of interferon genes (STING), thereby dampening antiviral signaling during viral infections. Moreover, TRIM18 stabilizes PPM1A by inducing K63-linked ubiquitination of PPM1A.

**Conclusions:**

Our results indicate that TRIM18 serves as a negative regulator of viral myocarditis, lung inflammation and brain damage by downregulating innate immune activation induced by both RNA and DNA viruses. Our data reveal that TRIM18 is a critical regulator of innate immunity in viral induced diseases, thereby identifying a potential therapeutic target for treatment.

**Supplementary Information:**

The online version contains supplementary material available at 10.1186/s12929-022-00840-z.

## Background

Innate immunity is the first line of defense against invading pathogens including RNA and DNA viruses. Activation of innate immunity requires the recognition of pathogen-associated molecular patterns (PAMPs), such as atypical viral nucleic acids, by pattern-recognition receptors (PRRs) on innate immune cells [1]. Recognition of PAMPs by PRRs activates signaling cascades leading to the production of type I interferon (IFN), which is central to host anti-viral defense by upregulating IFN-stimulated genes (ISGs) that limit virus dissemination and activate adaptive immune responses [2, 3]. Multiple PRRs have been identified that recognize viral RNA and DNA and induce type I IFN, including membrane-bound sensors such as Toll-like receptors (TLRs) [1, 4], cytosolic RNA sensors such as retinoic acid-inducible gene-I (RIG-I)-like receptors (RLRs) [5] and poly(ADP-ribose) polymerase 9 (PARP9) [6], and cytosolic DNA sensors such as cyclic GMP-AMP synthase (cGAS) [7], interferon gamma-inducible protein 16 (IFI16) [8] and DDX41 [9]. Cytosolic viral RNA is mainly detected by RLRs containing RIG-I and melanoma differentiation-associated gene 5 (MDA5) [10–12], which activate the downstream adaptor mitochondrial antiviral-signaling (MAVS, also known as IPS-1, CARDIF and VISA) [13–15]. MAVS then assembles a signaling platform that recruits TANK-binding kinase 1 (TBK1) and IκB-kinase-i (IKKi) kinases to phosphorylate the transcription factor IFN regulatory factor 3 (IRF3), leading to induction of type I IFN [16, 17]. Cytosolic DNA is mainly sensed by cGAS that activates the adaptor protein stimulator of interferon genes (STING) [2]. Similar to MAVS, the STING pathway converges on the recruitment of TBK1 to activate type I IFN production [17]. Although type I IFN production is essential for the host to restrict viral infection, aberrant and excessive type I IFN production results in inflammatory tissue injury and autoimmune diseases. Therefore, precise control of IFN production is critical for efficient viral clearance while avoiding harmful immunopathology [18].

Viruses such as severe acute respiratory syndrome coronavirus (SARS-CoV) and influenza virus are major threats to human health [19], since they can cause severe disease during an outbreak in humans. Viral myocarditis is an inflammation of the heart muscle resulting from viral infections and RNA virus Coxsackievirus B3 (CVB3) infection is the main cause of viral myocarditis [20, 21]. Herpes simplex encephalitis (HSE) is caused by DNA virus herpes simplex virus type I (HSV-1) in the brain. It is the most common cause of sporadic fatal encephalitis worldwide [22]. Viral pneumonia is an inflammation of the lungs caused by respiratory viruses [23, 24], such as DNA virus adenovirus, and RNA viruses including influenza virus, rhinovirus, and SARS-CoV-2 causing the pandemic of Coronavirus Disease 2019 (COVID-19) [25]. The ongoing COVID-19 pandemic is global challenge and demands a fundamental understanding of the mechanisms of viral pathogenicity and antiviral immunity. In our work with the tripartite motif (TRIM) family proteins, most of which have E3 ubiquitin ligase activities, we and others have identified various functions including antiviral innate immunity, intracellular signaling, development, apoptosis, protein quality control and autophagy [26]. TRIM18 is one member of TRIM family proteins and is encoded by Midline 1 gene on the X chromosome, whose mutations are linked to a rare genetic disease called X-linked Opitz G/BBB Syndrome (XLOS) [27]. Several studies in the mouse, Xenopus and chicken embryos highlight that TRIM18 also contributes to different neurodevelopmental events [28–31]. Furthermore, TRIM18 functions as a E3 ubiquitin ligase to inhibit protein phosphatase 2A activity, which promotes allergic asthma induced by allergen house dust mite (HDM) and rhinovirus [32]. Moreover, TRIM18 plays a crucial role in modulating the efficiency of the translation machinery with possible implications in neurodegeneration [33, 34]. TRIM18 has also been implicated in development of prostate cancer [35, 36], since TRIM18 can bind Androgen Receptor (AR) mRNA to regulate prostate cancer cell proliferation [36]. Despite those observations, detailed mechanisms by which TRIM18 controls viral pathogenicity and antiviral innate immunity remain unknown.

In the present study, we found that the E3 ligase TRIM18 served as a negative regulator of viral myocarditis, pneumonia, and encephalitis by downregulating innate immune activation against both RNA and DNA viruses. TRIM18 was shown to recruit protein phosphatase 1A (PPM1A) to dephosphorylate TBK1, which inactivates TBK1 and blocks its interactions with upstream adaptors MAVS and STING, thereby dampening type I IFN mediated antiviral signaling during viral infections. Given the critical role of TRIM18 in controlling viral pathogenicity and antiviral innate immunity against RNA and DNA viruses, TRIM18 may serve as an important therapeutic target for controlling viral-induced diseases including COVID-19.

## Methods

### Mice

*Trim18* knockout (KO) mice were originally generated as described previously [37] and cryorecovered from the Australian Phenomics Facility, Australian National University. All animals were on the C57BL/6 genetic background and maintained in the specific pathogen-free facility under 12 h light/dark cycle at 22–24 °C with unrestricted access to food and water for the durations of the experiments at Houston Methodist Research Institute in Houston, Texas. Animal use and care were ethically approved by the Houston Methodist Animal Care Committee, in accordance with institutional animal care and use committee guidelines.

### Reagents

The high molecular weight poly I:C (Cat: tlrl-pic), 5’triphosphate double-stranded RNA (5’pppRNA, Cat: tlrl-3prna), HSV-60 (Cat: tlrl-hsv60n), 2′3’-cGAMP (cGAMP, Cat: tlrl-nacga23), and LPS (Cat: tlrl-3pelps) were from Invivogen. Lipofectamine 3000 (Cat: L3000015) was from Invitrogen. The following antibodies were used for immunoblot analysis: anti-TRIM18 (IB:1:1000; MBS9127519; MyBioSource), anti-MAVS (IB:1:1000; #3993S; Cell Signaling Technology), anti-STING (IB:1:1000; #13647S; Cell Signaling Technology), anti-IRF3 (IB:1:1000; #4302S; Cell Signaling Technology), antibody to phosphorylated IRF3 at Ser396 (IB:1:1000; #4947S; Cell Signaling Technology), anti-TBK1 (IB:1:1000; #51872S; Cell Signaling Technology), antibody to phosphorylated TBK1 (IB:1:1000; #5483S, Cell Signaling Technology), anti-MAVS (IB:1:1000; #83000S; Cell Signaling Technology), anti-GAPDH (IB:1:10,000; G9295; Sigma), anti-β-actin (IB:1:10,000; A3854; Sigma), anti-HA (IB:1:5000; H6533; Sigma), anti-Myc (IB:1:5000; A5598; Sigma), peroxidase affinipure goat anti-mouse light chain specific IgG (IB:1:10,000; 115–035-174, Jackson ImmunoResearch), and peroxidase mouse anti-rabbit light chain specific IgG (IB:1:10,000; 211–032-171, Jackson ImmunoResearch). Anti-HA and anti-Myc agarose beads were from Sigma. Lentiviral vectors for shRNA were from Dharmacon Inc. (Horizon Discovery Group company): TRIM18 (clone TRCN0000019813); MAVS (clone TRCN0000146651); STING (clone TRCN0000161052). The IFN-β and IFN-α enzyme-linked immunosorbent assay (ELISA) kits were from PBL Interferon Source. The IL-6, TNF-α and IL-1β ELISA kits were from R&D systems. The Dual-Luciferase Reporter Assay System (E1910) was from Promega. Influenza A virus (PR8 A/Puerto Rico/8/1934(H1N1)), Coxsackievirus B3 (CVB3, strain Nancy), human adenovirus (Type 5) and herpes simplex virus type I (HSV-1, KOS strain) were from ATCC (ATCC® VR-95™, ATCC® VR-30™, ATCC®VR-1516™, and ATCC®VR-1493™).

### Cell culture

Human THP-1 cells (ATCC® TIB-202™) were differentiated to macrophages (THP-1 macrophages) with 60 nM phorbol 12-myristate 13-acetate (PMA; Sigma) for 16 h, and cells were cultured for an additional 48 h without PMA [6]. Bone marrow cells were isolated from the tibia and femur and cultured in RPMI1640 medium with 10% FBS, 1% penicillin–streptomycin, and 10% L929 conditioned media containing macrophage-colony stimulating factor (M-CSF) for 6 days to harvest bone marrow-derived macrophages (BMDM) [6, 38–40].

### Lentivirus transduction and stimulation

The pLKO.1 lentiviral vector carrying a scrambled shRNA or target gene sequences (Open Biosystems) were co-transfected into HEK 293FT cells (R70007; ThermoFisher Scientific) with packaging plasmids psPAX2 (Addgene 14858) and pMD2.G (Addgene 12259) using lipofectamine 3000 (ThermoFisher Scientific) for producing lentivirus. Human THP-1 macrophages were infected by lentivirus as previously [9, 41]. After 24 h of culture, cells were selected by the addition of puromycin (2 ng/ml) to the medium. The knockdown efficiency was detected with immunoblot analysis. The cells after lentivirus transduction were stimulated for the indicated time with 5’pppRNA (0.5 μg/ml), poly I:C (0.5 μg/ml), dsDNA from HSV-1 virus (HSV60, 2.5 μg/ml) and cGAMP (2.5 μg/ml) delivered by Lipofectamine 3000 or infection with RNA viruses including influenza A virus (influenza A virus PR8 strain, Flu PR8) and Coxsackievirus B3 (CVB3) or DNA viruses including adenovirus and HSV-1 at multiplicity of infection (MOI) of 2. The concentrations of IFN-α and IFN-β in culture supernatants were measured by ELISA.

### In vivo virus infection

For the in vivo CVB3 infection study, age-matched *Trim18*^+/+^ (WT) and *Trim18*^−/−^ (KO) male mice (*n* = 10 per strain, 6 weeks old) were injected intraperitoneally with CVB3 (1 × 10^7^ PFU/mouse). The survival of mice was monitored daily for 14 days after CVB3 infection. At day 2 after infection, mice were euthanized, and whole hearts were excised into PBS and homogenized for determining viral titers and concentrations of cytokines by ELISA. Additionally, the heart weight was determined by scale before and after CVB3 infection for 5 days.

For the in vivo influenza PR8 virus (Flu PR8) infection study, age- and sex-matched *Trim18*^+/+^ (WT) and *Trim18*^−/−^ (KO) mice (*n* = 10 per strain, 6 weeks old) were infected intranasally with Flu PR8 (1 × 10^5^PFU/mouse) in 50 µl PBS after anesthesia [38]. The survival of mice was monitored daily for 14 days after Flu PR8 infection. The viral titers in the lung were determined by standard plaque assays. To obtain bronchoalveolar lavage fluid (BALF) from mice, tracheas were cannulated after exsanguination and lungs were washed with 1 ml of PBS. BALF samples were centrifuged (800* g*, 5 min) to isolate cells and supernatants were centrifuged again (13,000* g*, 1 min) to completely remove remaining cells. The concentration of cytokines in BALF were measured by ELISA.

For the in vivo HSV-1 infection study, age- and sex-matched *Trim18*^+/+^ (WT) and *Trim18*^−/−^ (KO) mice (*n* = 10 per strain, 6 weeks old) were infected with HSV-1 (5 × 10^6^PFU/mouse) by intravenous injection [6, 39]. The survival of mice was monitored daily for 14 days after HSV-1 infection. Viral titers were measured by plaque assays using homogenates from brains of infected mice at 2 days post-infection. Sera were collected at 12-h post infection to measure the productions of IFN-α and IFN-β cytokines by ELISA.

For the in vivo adenovirus infection study, age- and sex-matched *Trim18*^+/+^ (WT) and *Trim18*^−/−^ (KO) mice (*n* = 10 per strain, 6 weeks old) were infected intranasally with adenovirus (1 × 10^8^PFU/mouse) in 50 µl PBS after anesthesia [39]. At day 2 after infection, mice were euthanized for collecting BALF from lung and then lung tissue collected for determining viral titers. The concentration of cytokines in BALF were measured by ELISA.

### Virus titration

After CVB3 or Flu PR8 infection in mice, total heart or lung tissues were removed and homogenized to prepare heart or lung extracts in 1 ml of PBS (pH 7.4). The supernatants from the homogenized heart or lung tissues were diluted and then used to infect confluent HeLa cells (ATCC® CCL-2™) or Madin-Darby canine kidney (MDCK) cells (ATCC® CCL-34™) cultured on 12-well plates, respectively. At 1 h post-infection, the supernatant was removed, and 2% low melting-point agarose was overlaid. At 3-days post-infection, the overlay was removed, and cells were fixed with methanol: acetic acid solution (3:1 of methanol: acetic acid) for 20 min and stained with 0.2% crystal violet. Plaques were counted, averaged, and multiplied by the dilution factor to determine viral titer [6, 38, 42–44].

After HSV-1 or adenovirus infection in mice, total brain or lung tissues were removed and homogenized to prepare brain or lung extracts in 1 ml of PBS (pH 7.4). The supernatants from the homogenized tissues were diluted and then used to infect confluent Vero cells (ATCC® CCL-81™) or A549 cells (ATCC® CCL-185™) cultured on 12-well plates, respectively. At 1 h post-infection, the supernatant was removed, and 2% low melting-point agarose was overlaid. At 3-days post-infection, the overlay was removed, and cells were fixed with methanol: acetic acid solution (3:1 of methanol: acetic acid) for 20 min and stained with 0.2% crystal violet as described above for the HeLa and MDCK cells. Plaques were counted, averaged, and multiplied by the dilution factor to determine viral titer [39, 45, 46].

### Quantification of cells numbers in Bronchoalveolar lavage fluid (BALF)

To obtain differential cell counts in BALF samples, a 100 µL aliquot of cells was subjected to cytocentrifugation (Cytospin; Cytopro Wescor; Syracuse, NY), air dried, and stained with a Hema 3 staining kit (Fisher Scientific) and a modified Wright–Giemsa stain [47]. Differential cell counts were made from a minimum count of 300 cells in light microscopy. Cell counts were thus expressed for total cells, macrophages, neutrophils, and lymphocytes.

### Echocardiography measurement

Transthoracic echocardiography was performed at day 4 after CVB3 inoculation. Mice were anesthetized by isoflurane inhalation. A comprehensive echocardiographic study was performed, including 2-dimensional imaging and M-mode imaging using the Vevo 2100 system (VisualSonics, Toronto, Canada) [48].

### Histology and histology score analysis

Hearts and brain were removed from mock and CVB3 virus or HSV-1 infected *Trim18*^+/+^ (WT) and *Trim18*^−/−^ (KO) mice respectively, while lungs were removed from mock as well as Flu PR8 or adenovirus infected *Trim18*^+/+^ (WT) and *Trim18*^−/−^ (KO) mice. These removed tissues were washed using PBS before being fixed with 10% formaldehyde for 24 h at room temperature. The tissues were embedded in paraffin and processed by standard techniques. Longitudinal 5-μm sections were stained with Hematoxylin & Eosin (H&E) [6, 38, 39, 44, 47, 49].

The severity of myocarditis in the hearts of CVB3 infected mice was scored as follows[50]: 0, no lesions; 1, one or few small lesions; 2, multiple small or few large lesions; 3, multiple small and large lesions; 4, massive lesions. Lesions were defined as areas of inflammation and/or cardiomyocyte necrosis and loss. The extent of pathologic inflammation in the brains of HSV-1 infected mice was scored as follows [51]: 0, no apparent inflammation, 1, minimal inflammation, 2 intermediate inflammation, 3 large inflammation. Inflammation was judged on the presence and extent of cellular infiltrate and reactive gliosis. The damage severity of pneumonia in the lungs of Flu PR8 or adenovirus infected mice was scored on a 5-point scale as follows [52]: 0 indicates none or very minor; 1 indicates mild; 2 indicates intermediate; 3 indicates moderately severe; and 4 indicates severe and widespread. Lesions were defined as areas of inflammation and/or cell necrosis and loss.

### Immunohistochemical analysis

For immunohistochemistry (IHC) staining, paraffin-embedded hearts were cut transversely into 5-μm sections. Following a 5-min high-pressure antigen retrieval process in citrate buffer with a pH of 6.0, the heart sections were blocked with 10% bovine serum albumin for 60 min and were subsequently incubated overnight at 4 °C with the primary antibodies including anti-neutrophil Rat antibody (ab2557, Abcam), anti-macrophage Rat antibody (ab56297, Abcam), anti-NK cell marker mouse antibody (sc-59340, Santa Cruz), anti-T cell CD3 Rabbit antibody (ab16669, Abcam), anti-TRIM21 Rabbit antibody (12108-1-AO, Proteintech) and anti-TRIM18 Rabbit antibody (MBS9127519, MyBioSource). Binding was visualized with the appropriate peroxidase-conjugated secondary antibodies (Horseradish Peroxidase AffiniPure Goat Anti-Rat or Anti-Rabbit IgG (H + L), 111-035-003, Jackson ImmunoResearch) for 30 min at 37 °C.

### In vitro co-immunoprecipitation and immunoblot analysis

For the preparation of purified TRIM18 and PPM1A, HEK293T cells were transfected with expression plasmids encoding full-length or truncated versions of HA- or Myc-tagged TRIM18 or PPM1A. Lysates were prepared from the transfected cells, followed by incubation with anti-HA or anti-Myc beads. Proteins were eluted from the beads after beads were washed six times with PBS. For precipitation with anti-HA or anti-Myc beads, purified HA-tagged wild-type PPM1A or truncations of PPM1A were incubated for 2 h with purified Myc-tagged TRIM18 or purified HA-tagged TRIM18 or truncations of TRIM18 were incubated for 2 h with purified Myc-tagged PPM1A. Beads were added; after 2 h of incubation, the bound complexes were pelleted by centrifugation. Proteins and beads were analyzed by immunoblot analysis with anti-HA or anti-Myc Abs. For immunoprecipitation of endogenous proteins, the whole-cell lysates of WT and TRIM18 KO BMDM that were either mock infected or infected with CVB3 or adenovirus were incubated with anti-PPM1A, anti-TBK1 or immunoglobulin G antibodies. After 2 h of incubation, the protein A/G beads were added for another 4 h incubation, and the bound complexes were pelleted by centrifugation. Proteins and beads were analyzed by immunoblot analysis with anti-TRIM18, anti-PPM1A, anti-TBK1, anti-MAVS, and anti-STING antibodies. WT and TRIM18 KO BMDM were either mock infected or infected with CVB3 or adenovirus for 2 h, and were then lysed in 1% NP-40 lysis buffer (50 mM Tris–HCl, 1%NP-40, 0.1% SDS, 150 mM NaCl) supplemented with protease inhibitor (ThermoFisher Scientific) followed by centrifugation. Supernatants were collected and incubated with SDS sample buffer by boiling of samples for 8 min followed by SDS-PAGE and immunoblot analysis. Immunoblot films were scanned by CanoScan 9000F mark II and images were processed with Adobe Photoshop Creative Cloud (CC) 2019 (version 20.0.10).

### Dual-luciferase reporter assay

HEK 293 T cells were seeded in 24-well plates and transfected the following day with the IFN-β firefly luciferase reporter vector (IFN-β-Luc, 50 ng), TK-renilla luciferase reporter vector (5 ng), Flag-MDA5 (100 ng), Flag-MAVS (50 ng), Flag-TBK1 (100 ng), Flag-IKKi (100 ng), Myc-cGAS (50 ng) and Myc-STING (50 ng) together with increasing doses of HA-TRIM18 plasmid (0, 100, 200 ng) or HA-vector by Lipofectamine 3000 transfection (ThermoFisher Scientific) as per the manufacturer’s instructions. At 24 h after transfection, cells were lysed and then measured using a dual-luciferase reporter assay system (Promega) according to the manufacturer’s instructions.

### Quantitative RT-PCR

RNA was isolated using the RNeasy Kit (Qiagen) according to the manufacturer’s instructions. The isolated RNA was used to synthesize cDNA with the iScript cDNA Synthesis Kit (Bio-Rad). The quantitative RT-PCR (qRT-PCR) was performed on a CFX-96 real-time PCR detection system (Bio-Rad) with iTaq Universal SYBR Green Supermix (Bio-Rad). All qRT-PCR primers were listed in Additional file 1: Table S3.

### Flow cytometry

Mouse spleen cells were isolated from *Trim18*^+/+^ (WT) and *Trim18*^−/−^ (KO) mice. The cells were then fixed and stained with Zombie Aqua fixable viability kit (423,102, Biolegend), FITC anti-mouse CD45 antibody (30-F11, Biolegend), Brilliant Violet 785 anti-mouse CD11b antibody (M1/70, Biolegend) and PE/Cyanine7 anti-mouse F4/80 antibody (BM8, Biolegend) (1 μl antibody for 2 million cells) for analyzing composition of CD11b^+^F4/80^+^ macrophages. Flow cytometry data were acquired on an LSR-II flow cytometer (Beckton Dickinson) and analyzed using FlowJo v10 software (Tree Start) [6, 38, 44].

### Metadata analysis

For metadata analysis, human TRIM18 expression was evaluated using the publicly accessible database Genecards (https://www.genecards.org/cgi-bin/carddisp.pl?gene=MID1&keywords=TRIM18). Mouse TRIM18 expression was evaluated from Immunological Genome Project (ImmGen) ULIRNASeq dataset (GSE127267) obtained using skyline data viewer from ImmGen website (http://rstats.immgen.org/Skyline/skyline.html).

### Statistical analysis

All samples sizes are large enough to ensure proper statistical analysis. Data are represented as the means ± DS of at least three experiments. Statistical analyses were performed using GraphPad Prism8 software (GraphPad Software, Inc.) and Microsoft Office Excel 2016. Statistical significance is calculated using Student’s two tailed unpaired t test. The log-rank (Mantel-Cox) test is used for survival comparisons. NS, not significant (p > 0.05); *p < 0.05; **p < 0.01; ***p < 0.001.

## Results

### TRIM18 downregulates type I IFN production in human macrophages in response to both RNA and DNA viruses

**Fig. 1:**
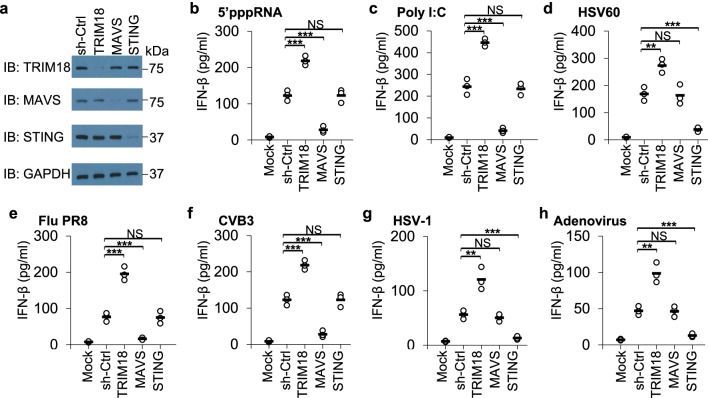
TRIM18 inhibits type I IFN production by human THP-1 macrophages in response to stimulations with dsRNA and dsDNA or infections with RNA and DNA viruses.** a** The immunoblot (IB) analysis of TRIM18, MAVS or STING expression in human THP-1 macrophages treated with shRNA to knockdown expression of TRIM18, MAVS or STING. A scrambled shRNA served as a control (sh-Ctrl) and the glyceraldehyde 3-phosphate dehydrogenase (GAPDH) served as the loading control. The position of protein markers (shown in kDa) is indicated at right. **b-h**, ELISA of IFN-β production from human THP-1 macrophages treated with the indicated shRNA after a 12 h stimulations with dsRNA including 5’pppRNA (0.5 μg/ml) **b** and poly I:C (0.5 μg/ml) **c** or dsDNA from HSV-1 virus (HSV60, 2.5 μg/ml) **d** delivered by Lipofectamine 3000, or 12 h infections with RNA viruses including influenza A virus (influenza A virus PR8 strain, Flu PR8) **e** and Coxsackievirus B3 (CVB3) (**f**), or DNA viruses including HSV-1 **g** and adenovirus **h** at an MOI of 2 (n = 3 per group). Each circle represents an individual independent experiment and small solid black lines indicate the average of triplicates for results in (**b**–**h**). Mock, scrambled shRNA-treated cells without stimulation. NS, not significant (p > 0.05), **p < 0.01, ***p < 0.001, and p value was calculated by unpaired two-tailed Student’s *t* test. Data are representative of three independent experiments

Previously, we screened all 70 members of the TRIM family proteins in the mouse alveolar macrophage cell line MH-S by a small-interfering-RNA approach and identified TRIM29 as a crucial negative regulator in antiviral innate immunity [38]. Meanwhile, we found another E3 ligase TRIM18 was a potential negative regulator in antiviral innate immunity. We next investigated the role of TRIM18 in antiviral innate immunity by knocking down TRIM18 via short hairpin RNA (shRNA) in human THP-1 macrophages. The TRIM18-targeting shRNA produced efficient knockdown of TRIM18 at the protein level (Fig. 1a). We then stimulated these cells with 5′-triphosphorylated RNA (5′pppRNA, the ligand of RIG-I-like receptors (RLRs)), dsRNA poly I:C (high molecular weight poly I:C, the ligand of RLRs) and dsDNA from HSV-1 virus (HSV60, the ligand of cytosolic DNA sensors), and measured type I IFN IFN-α and IFN-β by enzyme-linked immunosorbent assay (ELISA). As a positive control, knockdown of the key adaptor MAVS in RLRs signaling pathway via shRNA abrogated the production of IFN-β (Fig. 1b, c) and IFN-α (Additional file 2: Fig. S1a, b) in THP-1 macrophages stimulated by cytosolic 5’pppRNA and poly I:C. As a negative control, the production of IFN-β and IFN-α was not affected in STING-knockdown THP-1 macrophages (Fig. 1b, c) (Additional file 2: Fig. S1a, b), which confirmed a previous report showing that STING plays a critical role in DNA sensing but no role in RNA sensing [53]. In contrast, knockdown of TRIM18 markedly increased production of IFN-β (Fig. 1b, c) and IFN-α (Additional file 2: Fig. S1a, b) by THP-1 macrophages compared to cells treated with control shRNA (sh-Ctrl). We next determined whether TRIM18 controlled DNA sensing pathway in human THP-1 macrophages. As a result, STING knockdown led to significant reduction of IFN-β and IFN-α production in THP1 macrophages in response to dsDNA HSV60 (Fig. 1d and Additional file 2: Fig. S1c), while the knockdown of MAVS in THP1 macrophages had little effect on IFN-β and IFN-α production in response to dsDNA HSV60 (Fig. 1d and Additional file 2: Fig. S1c), which confirms a previous report showing that the RNA-sensing adaptor molecule MAVS is not required for cytokine production in response to cytosolic DNA [54]. However, knockdown of TRIM18 markedly increased production of IFN-β (Fig. 1d) and IFN-α (Additional file 2: Fig. S1c) by THP-1 macrophages compared to cells treated with control shRNA. Furthermore, knockdown of TRIM18 in THP1 macrophages had no effect of IFN-α and IFN-β production in response to LPS (Additional file 2: Fig. S1d, e). These data suggested that TRIM18 negatively regulates production of IFN-α and IFN-β in human THP-1 macrophages in response to cytosolic dsRNA and dsDNA.

To further assess the role of TRIM18 in antiviral innate immunity against virus infection, we analyzed protein expression of TRIM18 in normal tissues from GeneCards [55]. TRIM18 had high expression in brain, heart and lung, but was less expressed in most of other tissues (Additional file 2: Fig. S2a). Therefore, we investigated the role of TRIM18 in antiviral immune response in human THP1 macrophages by infection with two RNA viruses coxsackievirus B3 (CVB3, a RNA virus targeting heart for inducing viral myocarditis) and influenza A virus PR8 strain (Flu PR8, a RNA virus targeting lung to induce viral pneumonia), and two DNA viruses including herpes simplex virus type I (HSV-1, a DNA virus targeting brain for inducing viral encephalitis) and human adenovirus (a DNA virus targeting lung for viral pneumonia). As expected, knockdown of the key adaptor MAVS abrogated the production of IFN-β and IFN-α in THP-1 macrophages in response to RNA viruses Flu PR8 and CVB3 (Fig. 1e, f, Additional file 2: Fig. S2b, c), but not to DNA viruses HSV-1 and adenovirus (Fig. 1g, h, Additional file 2: Fig. S2d, e), while STING knockdown led to significant reduction of IFN-β and IFN-α production in THP1 macrophages after infection with DNA viruses HSV-1 and adenovirus (Fig. 1g, h, Additional file 2: Fig. S2d, e), but not with RNA viruses Flu PR8 and CVB3 (Fig. 1e, f, Additional file 2: Fig. S2b, c). However, knockdown of TRIM18 significantly increased production of IFN-β (Fig. 1e–h) and IFN-α (Additional file 2: Fig. S2b–e) by THP-1 macrophages compared to cells treated with control shRNA after infection with RNA viruses Flu PR8 and CVB3 and DNA virus HSV-1 (Fig. 1e–h, Additional file 2: Fig. S2b–e). Taken together, these data indicate that TRIM18 negatively regulates type I IFN production in human THP1 macrophages in response to stimulation with dsRNA and dsDNA or infection with both RNA and DNA viruses.

### TRIM18 negatively regulates innate immune response in mouse macrophages in response to RNA and DNA viruses

To further determine the role of TRIM18 in antiviral innate immunity in mice, we generated TRIM18 knockout (KO) mice. The *Trim18* gene deletion was confirmed by PCR assisted genotyping analysis (Additional file 2: Fig. S3a). We first analyzed TRIM18 gene expression in different mouse immune cells using the Immunological Genome Project (ImmGen) and found TRIM18 was indeed highly expressed in mouse macrophages including peritoneal macrophages, splenic macrophages, alveolar macrophages and microglia macrophages (Additional file 2: Fig. S3b). Next, we isolated mouse peritoneal macrophages (MF PC) and splenic macrophages (MF Sp) and detected high expression of TRIM18 in macrophages from wild-type (WT) mice, while deletion of TRIM18 expression was confirmed by immunoblot analysis (Additional file 2: Fig. S3c). We also investigated if TRIM18 expression was affected by RNA virus or DNA virus infection in mouse bone marrow-derived macrophages (BMDM). We found TRIM18 was induced at both RNA (Additional file 2: Fig. S3d) and protein (Additional file 2: Fig. S3e) levels in mouse BMDM after RNA or DNA virus infection and the induction of TRIM18 was much stronger in mouse BMDM by DNA viruses HSV-1 and adenovirus than that by RNA viruses Flu PR8 and CVB3 (Additional file 2: Fig. S3d, e). Furthermore, TRIM18 had high expression in lung, brain and heart, and low expression in intestine, liver and kidney from WT mice after DNA virus HSV-1 infection (Additional file 2: Fig. S3f). Additionally, KO of TRIM18 did not change expression of surface markers CD11b and F4/80 by flow cytometry (Additional file 2: Fig. S4), indicating that TRIM18 does not affect differentiation markers of mouse macrophages.

**Fig. 2:**
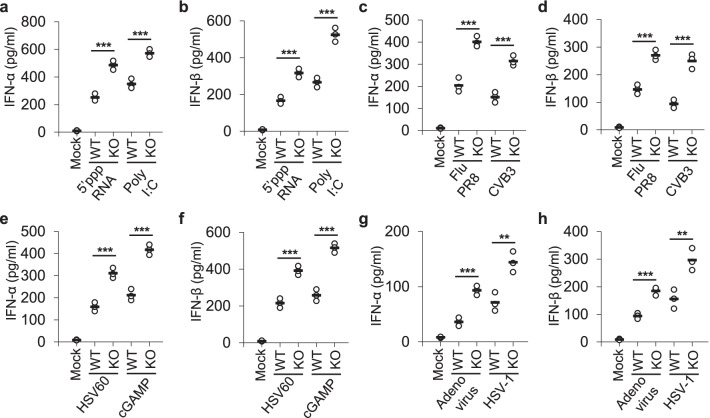
TRIM18 negatively regulates type I IFN production by BMDM upon stimulations of dsRNA and dsDNA or infections with RNA and DNA viruses.** a**–**d** ELISA of IFN-α **a**, **c** and IFN-β **b**, **d** production by BMDM from *Trim18*^+/+^ (WT) and *Trim18*^−/−^ (KO) mice after 12 h of stimulations with 5’pppRNA (0.5 μg/ml), poly I:C (0.5 μg/ml) delivered by Lipofectamine 3000 **a**, **b** or infections with RNA viruses including influenza A virus (influenza A virus PR8 strain, Flu PR8) and Coxsackievirus B3 (CVB3) **c**, **d** at an MOI of 2 (n = 3 per group). **e**–**h**, ELISA of IFN-α **e**, **g**) and IFN-β **f**, **h** production by BMDM from *Trim18*^+/+^ (WT) and *Trim18*^−/−^ (KO) mice after 12 h of stimulations with dsDNA from HSV-1 virus (HSV60, 2.5 μg/ml) and cGAMP (2.5 μg/ml) delivered by Lipofectamine 3000 **e**, **f** or infections with DNA viruses including adenovirus and HSV-1 **g**, **h** at an MOI of 2 (n = 3 per group). Each circle represents an individual independent experiment and small solid black lines indicate the average of triplicates for results. **p < 0.01, ***p < 0.001, and p value was calculated by unpaired two-tailed Student’s *t* test. Mock, wild-type BMDM without stimulation or infection. Data are representative of three independent experiments

To further investigate the role of TRIM18 in response to RNA viruses, we prepared mouse BMDM from WT and TRIM18 KO mice, and stimulated BMDM with dsRNA poly I:C and 5′-triphosphate RNA (5’pppRNA), and measured type I IFN proteins (IFN-α and IFN-β) by ELISA as well as mRNA levels of interferon stimulated gene 15 (ISG15) and ISG56 by qRT-PCR. The results showed that TRIM18 KO BMDM produced much more IFN-α and IFN-β proteins (Fig. 2a, b) and mRNAs of ISG15 and ISG56 (Additional file 2: Fig. S5a, b) than WT BMDM in response to 5’pppRNA and poly I:C stimulation. In addition, we employed two RNA viruses including Flu PR8 and CVB3 to investigate TRIM18 in response to RNA viruses in mouse BMDM. BMDM from WT and TRIM18 KO mice were isolated and infected with RNA viruses Flu PR8 and CVB3. Compared with WT BMDM, TRIM18 KO BMDM produced 2- to threefold more IFN-α and IFN-β proteins (Fig. 2c, d) and twofold more mRNAs of ISG15 and ISG56 (Additional file 2: Fig. S5c, d) post-infection by RNA viruses Flu PR8 and CVB3. Collectively, these data demonstrate a negative role for TRIM18 in regulating production of type I IFN and ISGs in mouse macrophages in response to dsRNA and RNA viruses.

To further determine the role of TRIM18 in response to DNA viruses, we prepared mouse BMDM from WT and TRIM18 KO mice, and stimulated BMDM with dsDNA HSV60 and cGAMP (STING stimulator in DNA sensing pathway), and measured type I IFN proteins by ELISA and mRNA levels of ISG15 and ISG56 by RT-qPCR. Consistent with the earlier results, TRIM18 KO BMDM produced much more IFN-α and IFN-β proteins (Fig. 2e, f) and mRNAs of ISG15 and ISG56 (Additional file 2: Fig. S5e, f) than WT BMDM in response to HSV60 and cGAMP stimulation. Furthermore, two DNA viruses including HSV-1 and human adenovirus were chosen to investigate role of TRIM18 in response to DNA viruses in mouse BMDM. BMDM from WT and TRIM18 KO mice were prepared and infected with DNA viruses adenovirus and HSV-1. The results showed TRIM18 KO BMDM produced significantly more IFN-α and IFN-β proteins (Fig. 2g, h) and mRNAs of ISG15 and ISG56 (Additional file 2: Fig. S5g, h) than WT BMDM after infection with DNA viruses. Taken together, these data suggest that TRIM18 is a negative regulator of type I IFN and ISGs productions in mouse macrophages in response to dsDNA and DNA viruses.

### Deletion of TRIM18 protects mice from viral myocarditis

**Fig. 3:**
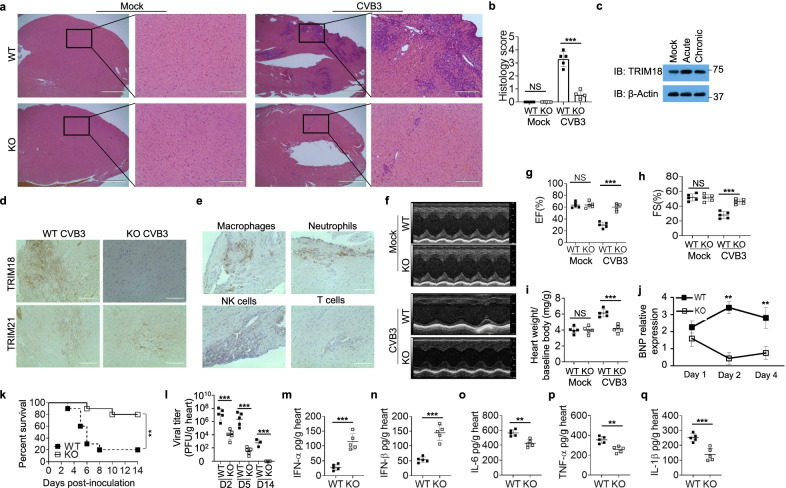
Deletion of TRIM18 protects mice from myocarditis induced by RNA virus CVB3 in vivo. **a** Hematoxylin and eosin (H&E)-staining of heart sections from age-matched *Trim18*^+/+^ (WT) and *Trim18*^−/−^ (KO) male mice after intraperitoneal infection with CVB3 (1 × 10^7^ PFU per mouse) for 4 days. Scale bars represent 1000 μm for original images and 200 µm for enlarged images. **b** Histology score analysis of viral myocarditis in heart sections from mice as in (**a**). **c** Immunoblot (IB) analysis of TRIM18 expression in hearts from WT mice without or with intraperitoneal CVB3 acute infection (1 × 10^7^ PFU per mouse) or chronic infection (1 × 10^3^ PFU per mouse) for 2 days. The position of protein markers (shown in kDa) is indicated at right. **d** Immunohistochemistry (IHC) analysis of TRIM18 and TRIM21 expression in hearts from WT and KO male mice after CVB3 infection. **e** IHC analysis of the infiltrated cells in CVB3 infected hearts using anti-macrophage marker antibody, anti-neutrophil marker antibody, anti-NK cell marker antibody and anti-T cell marker antibody, respectively. Scale bars represent 100 μm in (**d, e**). **f** Representative M-mode images of hearts from WT and KO male mice at day 4 after CVB3 infection by echocardiography analysis. **g**–**h** Cardiac function analysis of ejection fraction (EF) **g** and fractional shortening (FS) **h** of hearts from mice as in (**f**) (n = 5 per group). **i** The assessment of heart weight/baseline body weight from WT and KO male mice (n = 5 per group) at day 0 or day 6 after CVB3 infection. **j** The qRT-PCR analysis of brain natriuretic peptide (BNP) mRNA in the heart of from WT and KO male mice (n = 5 per group) at day 1, day 2 or day 4 after CVB3 infection.; results are presented relative to those of mock mice. **k** Survival of age-matched WT and KO male mice after intraperitoneal infection with CVB3 (1 × 10^7^ PFU per mouse) (n = 10 per group). **l** Viral titers in homogenates of hearts from WT and KO male mice at day 2 (D2), day 5 (D5) and day 14 (D14) after CVB3 infection (n = 5 per group for D2 and D5, n = 3 per group for D14). **m**–**q**, ELISA of IFN-α (**m**), IFN-β (**n**) IL-6 (**o**), TNF-α (**p**) and IL-1β **q** in hearts from mice as in **k** (n = 5 per group). Error bars indicate standard error of the mean for results in (**b**, **g**–**j, l**–**q**). NS, not significant (p > 0.05), **p < 0.01, ***p < 0.001, and p value was calculated by unpaired two-tailed Student’s *t* test and Gehan-Breslow-Wilcoxon test for survival analysis. Data are representative of three independent experiments

Viral myocarditis has been recognized as a cause of congestive heart failure and CVB3 infection is the main cause of viral myocarditis [20, 21]. Since TRIM18 is highly expressed in heart and TRIM18 negatively regulates innate immune response to RNA virus CVB3 in macrophages, we investigated the functional importance of TRIM18 in controlling CVB3 induced myocarditis in vivo. We first intraperitoneally infected both WT and TRIM18 KO mice with the RNA virus CVB3 and checked cardiac histology and functions. The heart histopathology revealed TRIM18 KO mice had significantly reduced cardiac inflammation and infiltration of inflammatory cells compared with WT mice following CVB3 infection (Fig. 3a, b). Additionally, TRIM18 expression was induced in hearts of WT mice with CVB3 infection (Fig. 3c), while TRIM18 induction was stronger in hearts of mice during CVB3 acute infection than that during CVB3 chronic infection (Fig. 3c). It’s reported that another TRIM family member TRIM21 could restrict CVB3 induced cardiac injury by positively regulate IRF3-mediated type I IFN production [68]. We then compared the expressions of TRIM18 and TRIM21 in hearts from WT and TRIM18 KO mice after CVB3 infection. The immunohistochemistry (IHC) data showed that there was high expression of TRIM21 in hearts from WT and TRIM18 KO mice after CVB3 infection (Fig. 3d). However, TRIM18 had higher expression than TRIM21 in heart from CVB3 infected WT mice, while TRIM18 expression was gone in heart from TRIM18 KO mice after CVB3 infection (Fig. 3d). Furthermore, IHC data showed that there were major macrophages and neutrophils, and minor NK cells and T cells in the infiltrated cells in hearts from mice after CVB3 infection for two days (Fig. 3e). In agreement, the echocardiography of WT mice revealed impaired cardiac function (Fig. 3f) as evidenced by decreased ejection fraction (EF) (Fig. 3g) and fractional shortening (FS) (Fig. 3h) when compared with TRIM18 KO mice. Compared to WT mice, TRIM18 KO mice had less heart weight increase during viral myocarditis (Fig. 3i), a marker of cardiac inflammatory edema. Additionally, brain natriuretic peptide (BNP), a marker of heart failure, was dramatically reduced in TRIM18 KO hearts compared with WT hearts (Fig. 3j). Importantly, we found that most of WT mice succumbed to CVB3 infection, while the survival of TRIM18 KO mice was significantly better than that of their WT littermates (Fig. 3k). These data suggested that deficiency of TRIM18 could protect mice from CVB3 induced myocarditis by reducing cardiac inflammation with improved function. To further investigate the mechanisms by which TRIM18 knockout mice reduced CVB3 induced myocarditis, we next checked viral replication and type I IFN protein levels in heart homogenates by plaque-forming assay and ELISA, respectively. We found that the CVB3 viral loads were significantly reduced in hearts from TRIM18 KO mice compared with WT mice at day 2 (D2), day 5 (D5) and day 14 (D14) after CVB3 infection (Fig. 3l). Furthermore, TRIM18 KO mice had higher concentrations of IFN-α (Fig. 3m) and IFN-β (Fig. 3n) in the hearts than did their WT littermates after infection with CVB3. However, WT mice had higher cardiac inflammatory cytokines IL-6 (Fig. 3o), TNF-α (Fig. 3p) and IL-1β (Fig. 3q) than did their TRIM18 KO littermates after CVB3 infection. These data indicate that deletion of TRIM18 protects mice from CVB3 induced myocarditis by improving cardiac function and promoting innate immune activation.

### Knockout of TRIM18 protects mice from pneumonia and lung injury induced by viral infections

**Fig. 4:**
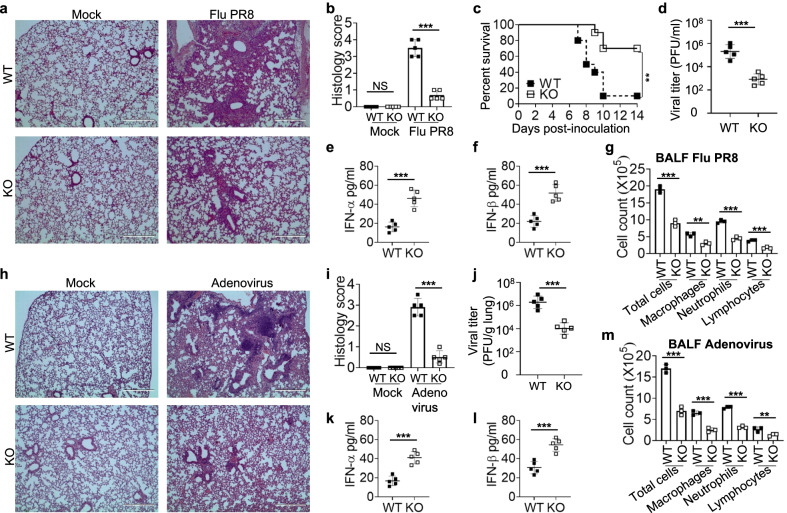
Knockout of TRIM18 protects mice from pneumonia and lung injury induced by RNA virus Flu PR8 or DNA virus adenovirus in vivo.** a** Hematoxylin and eosin (H&E)-staining of lung sections from age- and sex-matched *Trim18*^+/+^ (WT) and *Trim18*^−/−^ (KO) mice left infected (Mock) or infected for 4 days by intranasal infection of Flu PR8 virus (1 × 10^5^ PFU per mouse). **b** Histology score analysis of viral pneumonia in lung sections from mice as in (**a**). **c** Survival of age- and sex-matched WT and KO mice after intranasal infection with Flu PR8 virus (1 × 10^5^ PFU per mouse) (n = 10 per group). **d** Plaque assay of Flu PR8 virus titers in the lung of WT and KO mice infected for 2 days by intranasal infection of Flu PR8 virus (n = 5 per group). **e**–**f**, ELISA of and IFN-α **e** and IFN-β **f** in BALF samples from mice (n = 5 per group) as in (**d**). **g** Quantification of cell numbers in BALF samples from mice (n = 3 per group) as in (**d**). **h** Hematoxylin and eosin (H&E)-staining of lung sections from WT and KO mice left infected (Mock) or infected for 4 days by intranasal infection of adenovirus (1 × 10^8^ PFU per mouse). **i**, Histology score analysis of viral pneumonia in lung sections from mice as in (**h**). **j** Viral titers in homogenates of lung from WT and KO mice (n = 5 per group) at day 2 of intranasal infection with adenovirus (1 × 10^8^ PFU per mouse). **k**, **l** ELISA of IFN-α **k** and IFN-β **l** in BALF samples from mice as in **j** (n = 5 per group). **m** Quantification of cell numbers in BALF samples from mice (n = 3 per group) as in (**j**). Scale bars represent 400 μm for images in **a** and (**h**). Error bars indicate standard error of the mean for results in (**b**, **d–g**, **i–m**). NS, not significant (p > 0.05), **P < 0.01 and ***P < 0.001, and p value was calculated by unpaired two-tailed Student’s *t* test and Gehan-Breslow-Wilcoxon test for survival analysis. Data are representative of three independent experiments

Viral pneumonia is an inflammation of the lungs caused by respiratory viruses, such as influenza virus, adenovirus and SARS-CoV-2 causing the ongoing pandemic of COVID-19 [23, 24]. Interestingly, the public GEO profile database show that patients with SARS-CoV-2 infection have higher expression of TRIM18 (Additional file 2: Fig. S6), we hypothesize that TRIM18 may play crucial roles in controlling pneumonia and lung injury induced by respiratory viruses including SARS-CoV-2. As shown above, TRIM18 was highly expressed in lung and TRIM18 downregulated the innate immune responses to respiratory viruses including RNA virus influenza virus and DNA virus adenovirus in both human and mouse macrophages. Therefore, we investigated if TRIM18 could control viral pneumonia induced by those respiratory viruses including influenza virus and adenovirus in vivo. First, we infected both WT and TRIM18 KO mice intranasally with respiratory RNA virus influenza virus and checked lung inflammation and injury by histology. Indeed, lung histopathology revealed edema, alveolar hemorrhaging, alveolar wall thickness and neutrophil infiltration in lungs from TRIM18 KO mice were less marked than those from WT after influenza virus infection (Fig. 4a, b). Importantly, we found that TRIM18 KO mice were more resistant to influenza virus infection than their WT littermates (Fig. 4c). These results suggested that knockout of TRIM18 protected mice from lung injury and inflammation induced by RNA virus influenza virus in vivo. We further investigated the mechanisms by which deletion of TRIM18 protected mice from pneumonia infected by influenza virus. We determined viral amplification in lungs at day 2 post-infection by plaque-forming assay. We detected significantly less influenza virus loads in TRIM18 KO mice than in their WT littermates (Fig. 4d). We then detected type I IFN production in lungs by ELISA. Compared with WT mice, TRIM18 KO mice produced significantly more IFN-α (Fig. 4e) and IFN-β (Fig. 4f) following influenza virus infection. Additionally, there were increased infiltration cells mainly containing macrophages, neutrophils, and lymphocytes in bronchoalveolar lavage fluid (BALF) of WT mice with Flu PR8 infection, which were dramatically reduced in TRIM18 KO mice (Fig. 4g). These data indicate that TRIM18 deficiency protects mice from pneumonia induced by RNA virus influenza virus through restricting viral replication and promoting innate immune activation in vivo.

Next, we evaluated the importance of TRIM18 in controlling pneumonia following infection by respiratory DNA virus adenovirus in vivo. Both WT and TRIM18 KO mice were infected intranasally with adenovirus, which is normally transmitted by the nasal route and targets the lungs for pneumonia. Lung histopathology revealed much-reduced edema, alveolar hemorrhage, alveolar wall thickness, and neutrophil infiltrations in TRIM18 KO mice compared to the lung pathology in WT mice (Fig. 4h, i), indicating the importance of TRIM18 in promoting adenovirus induced pneumonia and lung inflammation. To further investigate the underlying mechanisms, we measured adenovirus replication and type I IFN production in lung by plaque-forming assay and ELISA, respectively. We found that the adenovirus loads were dramatically reduced in TRIM18 KO mice compared with WT mice (Fig. 4j). Additionally, there was significantly more IFN-α (Fig. 4k) and IFN-β (Fig. 4l) in the BALF from TRIM18 KO mice than that from WT mice at day 2 post infection. Compared with TRIM18 KO mice, there were much more infiltration cells mainly consisted of macrophages, neutrophils, and lymphocytes in BALF of WT mice with adenovirus infection (Fig. 4m). Collectively, these findings demonstrate that knockout of TRIM18 protects mice from pneumonia and lung injury induced by viral infections through enhancing activation of innate immunity in vivo.

### Deficiency of TRIM18 protects mice from encephalitis induced by HSV-1 infection

**Fig. 5:**
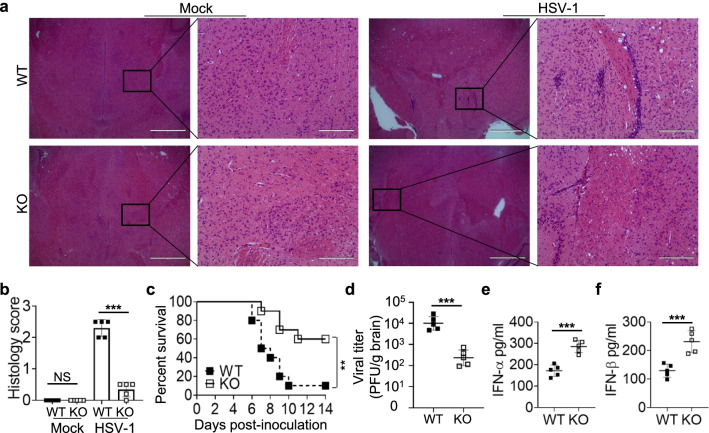
Deficiency of TRIM18 protects mice from brain damage induced by DNA virus HSV-1 in vivo. **a** Hematoxylin and eosin (H&E)-staining of brain sections from *Trim18*^+/+^ (WT) and *Trim18*^−/−^ (KO) mice left infected (Mock) or infected for 4 days by intravenous infection of HSV-1 (1 × 10^7^ PFU per mouse). Scale bars represent 1000 μm for original images and 200 µm for enlarged images. **b** Histology score analysis of viral encephalitis in brain sections from mice as in (**a**). **c** Survival of WT and KO mice (n = 10 per group) after intravenous injection of HSV-1 (1 × 10^7^ PFU per mouse). **d** Viral titers in homogenates of brains from WT and KO mice (n = 5 per group) after intravenous injection of HSV-1 (1 × 10^7^ PFU per mouse). **e**, **f**, ELISA of IFN-α **e** and IFN-β **f** in serum obtained from WT and KO mice (n = 5 per group) at 12 h after intravenous injection of HSV-1. Error bars indicate standard error of the mean for results in (**b**, **d**–**f**). NS, not significant (p > 0.05), **P < 0.01 and ***P < 0.001, and p value was calculated by unpaired two-tailed Student’s *t* test and Gehan-Breslow-Wilcoxon test for survival analysis. Data are representative of three independent experiments.

Herpes simplex encephalitis (HSE) is caused by HSV-1 infection of the brain and is the most common cause of sporadic fatal encephalitis worldwide[22]. Given that the high expression of TRIM18 in brain and the critical role of TRIM18 in regulating innate immune response to HSV-1 in macrophages, we further investigated if TRIM18 plays role in controlling brain damage and inflammation induced by DNA virus HSV-1 in vivo. We challenged WT and TRIM18 KO mice intravenously with HSV-1 virus and checked brain damage and inflammation by histology. The brain histopathology revealed much-reduced demyelination, necrosis, and inflammatory cell infiltration in TRIM18 KO mice as compared to the brain pathology in WT mice after HSV-1 infection (Fig. 5a, b). Compared with WT mice, TRIM18 KO mice had significantly higher survival rates (Fig. 5c). These results indicated that knockout of TRIM18 protected mice from brain damage and inflammation induced by HSV-1 virus in vivo. To further investigate the underlying mechanisms, we measured HSV-1 replication in brain and type I IFN production in serum by plaque-forming assay and ELISA, respectively. We detected significantly less HSV-1 virus in brain of TRIM18 KO mice than in WT mice on day 2 after infection (Fig. 5d). Moreover, TRIM18 KO mice had higher concentrations of IFN-α (Fig. 5e) and IFN-β (Fig. 5f) in the serum than did their WT littermates after HSV-1 infection. Collectively, these data demonstrate deficiency of TRIM18 protects mice from encephalitis induced by HSV-1 by enhancing innate immune activation.

### TRIM18 recruits PPM1A to inactivate TBK1 blocking TBK1 from interactions with its upstream adaptors during virus infection

**Fig. 6:**
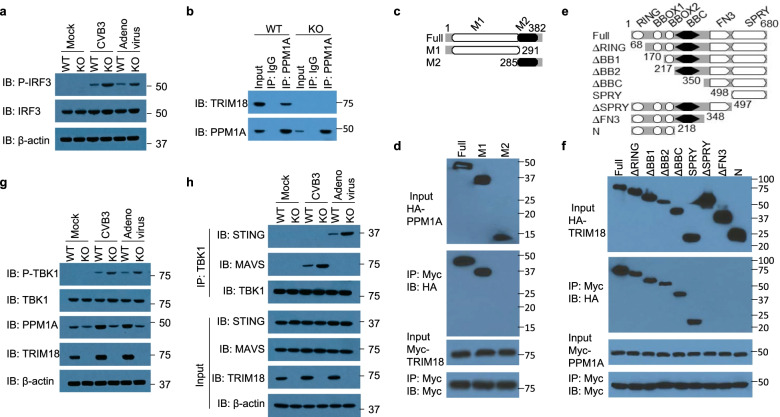
TRIM18 recruits PPM1A to dephosphorylate TBK1 for its inactivation and black the interactions of TBK1 with its upstream adaptors. **a** Immunoblot (IB) analysis of total and phosphorylated (p-) IRF3 as well as β-actin in lysates of WT and TRIM18 KO BMDM infected for 1 h without (Mock) or with CVB3 or adenovirus at an MOI of 5. **b** Immunoblot analysis of endogenous proteins TRIM18 and PPM1A precipitated with anti-PPM1A, or immunoglobulin G (IgG) from whole-cell lysates of WT and TRIM18 KO BMDM. **c** Schematic diagram showing full-length PPM1A (Full) and serial truncations of PPM1A with deletion of various domain (left margin); numbers at ends indicate amino acid positions (top). M1, the catalytic domain; M2, the C-terminal no catalytic domain. **d** Immunoblot analysis of purified HA-tagged full-length PPM1A and serial truncations of PPM1A with deletion of various domains alone with anti-HA antibody (top blot) or after incubation with Myc-tagged TRIM18 and immunoprecipitation with anti-Myc antibody (second blot), and immunoblot analysis of purified Myc-tagged TRIM18 with anti-Myc antibody (third blot) or after incubation with Myc-tagged TRIM18 and immunoprecipitation with anti-Myc antibody (bottom blot). **e** Schematic diagram showing full-length TRIM18 (Full) and serial truncations of TRIM18 with deletion (Δ) of various domain (left margin); numbers at ends indicate amino acid positions (top). RING, the really interesting new gene domain; BBOX, the B-box zinc-finger domain; BBC, the B-box C-terminal domain; FN3, the fibronectin type 3 domain; SPRY, the Sp1A kinase and Ryanodine receptors domain. **f** Immunoblot analysis of purified HA-tagged full-length TRIM18 and serial truncations of TRIM18 with deletion of various domains alone with anti-HA antibody (top blot) or after incubation with Myc-tagged PPM1A and immunoprecipitation with anti-Myc antibody (second blot), and immunoblot analysis of purified Myc-tagged PPM1A with anti-Myc antibody (third blot) or after incubation with Myc-tagged PPM1A and immunoprecipitation with anti-Myc antibody (bottom blot). **g** Immunoblot analysis of total and phosphorylated (p-) TBK1, PPM1A, TRIM18 as well as β-actin in lysates of WT and TRIM18 KO BMDM infected for 1 h without (Mock) or with CVB3 or adenovirus at an MOI of 5. **h** Immunoblot analysis of endogenous proteins STING, MAVS, TBK1 and TRIM18 from whole-cell lysates (Input) or precipitated with anti-TBK1 antibody (IP: TBK1) from lysates of WT and TRIM18 KO BMDM left infected (Mock) or infected with CVB3 or adenovirus at MOI of 10 for 6 h. The position of protein markers (shown in kDa) is indicated on the right. Data are representative of three independent experiments

To determine the molecular mechanisms by which the enhanced production of type I IFN and innate immune activation were achieved in BMDM from TRIM18 KO mice, we isolated BMDM from WT and TRIM18 KO mice and infected the cells without or with CVB3 and adenovirus for 1 h, then assessed activation of the transcription factors IRF3 by immunoblot analysis. We found that phosphorylation of IRF3 in TRIM18 KO BMDM was enhanced relative to WT BMDM after infection with CVB3 or adenovirus (Fig. 6a). Typically, cytosolic RNA or DNA is sensed by RIG-I-like receptors or DNA sensor such as cGAS, which then activate downstream MAVS and STING, respectively. MAVS or STING recruits downstream TBK1 to phosphorylate and activate IRF3 for inducing type I IFN. To further dissect the role of TRIM18 in these different IFN-I induction pathways, we examined the effect of TRIM18 on the IFN-β luciferase reporter activated by these components including MDA5, MAVS, TBK1, IKKi and cGAS/STING. Overexpression of TRIM18 reduced the IFN-β luciferase reporter activation by MDA5 and MAVS and to a greater extent by TBK1 and cGAS/STING (Additional file 2: Fig. S7a–d). However, TRIM18 did not inhibit downstream IKKi dependent IFN-β luciferase reporter activation (Additional file 2: Fig. S7e), indicating that TRIM18 targets the pathway at nodes between TBK1 and IKKi. To further investigate how TRIM18 regulates such signaling molecules, we used immunoprecipitation with an antibody specific to TRIM18 to identify TRIM18-interacting proteins in lysates of BMDM, followed by protein sequencing by liquid chromatography–mass spectrometry. Among a group of TRIM18-interacting proteins, we identified protein phosphatase, magnesium-dependent 1A (PPM1A; formerly called PP2C) (Additional file 1: Table S1). PPM1A has previously been reported to silence cytosolic RNA sensing and antiviral defense through direct dephosphorylation of TBK1 [56]. These collective data suggested that TRIM18 might recruit PPM1A to target and dephosphorylate TBK1 for dampening type I IFN production.

Next, we investigated if TRIM18 could interact with PPM1A in BMDM at the endogenous protein level. The anti-PPM1A antibody, but not control IgG, precipitated TRIM18 in WT BMDM, but not in TRIM18 KO BMDM (Fig. 6b), indicating real interaction between TRIM18 and PPM1A in resting BMDM. To further map the binding sites between TRIM18 and PPM1A, we analyzed interactions among Myc-tagged recombinant TRIM18 and HA-tagged recombinant full-length PPM1A, as well as truncation mutants of PPM1A (Fig. 6c). Both full-length PPM1A and the M1 catalytic domain of PPM1A bound to TRIM18 (Fig. 6d). Additionally, the mapping results for Myc-tagged recombinant PPM1A and HA-tagged full-length TRIM18 and their truncation mutants (Fig. 6e) showed that the C-terminal SPRY (Sp1A kinase and Ryanodine receptors) domain of TRIM18 bound to PPM1A (Fig. 6f). To further investigate whether TRIM18 recruits PPM1a to target and dephosphorylate TBK1, we isolated BMDM from WT and TRIM18 KO mice and infected the cells without or with CVB3 and adenovirus for 1 h, then assessed phosphorylation of TBK1 by immunoblot analysis. We found that phosphorylation of TBK1 in TRIM18 KO BMDM was significantly enhanced relative to that in WT BMDM after infection with CVB3 or adenovirus (Fig. 6g), suggesting that TRIM18 indeed recruited PPM1A to dephosphorylate TBK1 for TBK1 inactivation. Additionally, we found there was more PPM1A expression in WT BMDM than TRIM18 KO BMDM without and with CVB3 or adenovirus infection (Fig. 6b, g). To further investigate the outcomes of the recruitment and stability of PPM1A by TRIM18, we isolated BMDM from WT and TRIM18 KO mice and infected the cells without or with CVB3 and adenovirus for 5 h, then evaluated the interaction between TBK1 and its upstream adaptors MAVS or STING by co-immunoprecipitation and immunoblot analysis. We found that there was no interaction between TBK1 and MAVS or STING in BMDM from either WT or TRIM18 KO mice that had not been infected (Fig. 6h). However, after infection with CVB3 or adenovirus, interaction between TBK1 and MAVS or STING in both WT and TRIM18 KO BMDM was evident, but significantly enhanced in the TRIM18 KO BMDM relative to levels in WT BMDM (Fig. 6h), indicating that the recruitment of PPM1A by TRIM18 blocked interaction between TBK1 and its upstream adaptors MAVS or STING in macrophages after virus infection. Collectively, these data suggest that TRIM18 recruits PPM1A to dephosphorylate TBK1 for its inactivation and blocks interaction of TBK1 with its upstream adaptors MAVS and STING for signal transduction during virus infection.

### TRIM18 stabilizes PPM1A by mediating K63-linked ubiquitination

**Fig. 7:**
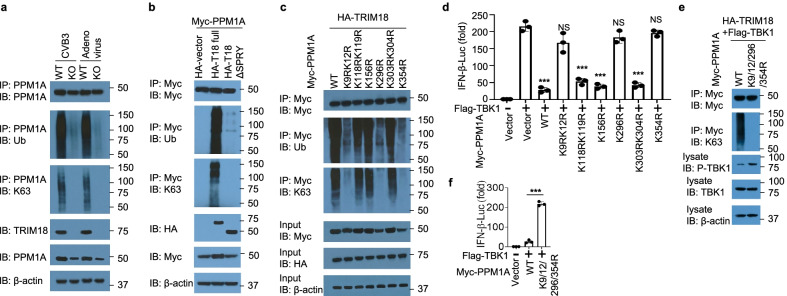
TRIM18 induces ubiquitination of PPM1A by K63 linkage. **a** Immunoblot (IB) analysis of TRIM18 (fourth blot), PPM1A (fifth blot) and β-actin (bottom blot) in WT and TRIM18 KO BMDM, the abundance (top blot), total ubiquitination (second blot), and K63-mediated ubiquitination (third blot) of PPM1A in those cells, infected for 4 h with CVB3 or adenovirus at an MOI of 5, assessed after immunoprecipitation with anti-PPM1A antibody. **b** Immunoblot analysis (with anti-Myc) of the abundance (top), total ubiquitination (second blot), and K63-linked ubiquitination (third blot) of Myc-tagged PPM1A in HEK293T cells transfected with empty vector or expression vector for full length HA-tagged TRIM18 (HA-T18 full), truncation T18-∆SPRY (losing binding site of PPM1A), assessed after immunoprecipitation with anti-Myc antibody; immunoblot analysis of whole-cell lysates with anti-HA (fourth blot), anti-Myc (fifth blot) and anti-β-actin (bottom). **c** Immunoblot analysis (with anti-Myc) of the abundance (top), total ubiquitination (second blot), and K63-linked ubiquitination (third blot) of Myc-tagged PPM1A in HEK293T cells transfected with HA-TRIM18 and Myc-PPM1A wild-type (WT) and its mutations including K9RK12R, K118RK119R, K156R, K296R, K303RK304R, and K354R, assessed after immunoprecipitation with anti-Myc antibody; immunoblot analysis of whole-cell lysates with anti-Myc (fourth blot), anti-HA (fifth blot) and anti-β-actin (bottom). **d** Luciferase assay in HEK293T cells transfected with IFN-β reporter, Flag-tagged plasmid expressing TBK1 along with Myc-tagged empty vector or PPM1A wild-type (WT) and its mutations including K9RK12R, K118RK119R, K156R, K296R, K303RK304R, and K354R. *Renilla* luciferase RL-TK was used as the internal control. **e** Immunoblot analysis (with anti-Myc) of the abundance (top), and K63-linked ubiquitination (second blot) of Myc-tagged PPM1A in HEK293T cells transfected with HA-TRIM18, Flag-TBK1 and Myc-PPM1A wild-type (WT) and its ubiquitination losing mutant K9/12/296/354R, assessed after immunoprecipitation with anti-Myc antibody; immunoblot analysis of whole-cell lysates with anti-phosphorylated (p-) TBK1 (third blot), anti-TBK1 (fourth blot) and anti-β-actin (bottom). **f** Luciferase assay in HEK293T cells transfected with IFN-β reporter, Flag-tagged plasmid expressing TBK1 along with Myc-tagged empty vector or PPM1A wild-type (WT) and its ubiquitination losing mutant K9/12/296/354R. *Renilla* luciferase RL-TK was used as the internal control. NS, not significant (p > 0.05), ***p < 0.001, and p value was calculated by unpaired two-tailed Student’s t test. The position of protein markers (shown in kDa) is indicated on the right. Data are representative of three independent experiments

Because TRIM18 is an E3 ubiquitin ligase and less PPM1A is seen in TRIM18 KO cells, we surmised that TRIM18 may mediate the stability of PPM1A. Consequently, we next determined whether TRIM18 was responsible for the ubiquitination of PPM1A ex vivo. We isolated BMDM from WT and TRIM18 KO mice and infected those cells with CVB3 or adenovirus for 6 h. Cell lysates were then prepared and analyzed for the ubiquitination of PPM1A. As results, PPM1A was modified via K63-mediated ubiquitination in BMDM from WT mice but not TRIM18 KO mice (Fig. 7a). Additionally, there was more PPM1A expression in WT BMDM that TRIM18 KO BMDM (Fig. 7a), which demonstrated the crucial role of K63-mediated ubiquitination in mediating stability of PPM1A. To investigate whether the ubiquitination of PPM1A was dependent on the binding site of TRIM18 with PPM1A, we transfected the HEK293T cells to co-express Myc-tagged PPM1A and HA-tagged full-length TRIM18, or truncated TRIM18 lacking the binding site of PPM1A (T18-∆SPRY). We then prepared cell lysates and incubated them for 5 min at 90 °C with 1% SDS (sodium dodecyl sulfate) to disrupt protein–protein interactions, followed by immunoprecipitation of Myc-tagged PPM1A. Immunoblot analysis of HA or ubiquitin demonstrated that the ubiquitination of PPM1A was strongly enhanced by overexpression of TRIM18 but not by overexpression of T18-∆SPRY (Fig. 7b). Immunoblot analysis of K63-linked ubiquitin further demonstrated that TRIM18 induced ubiquitination of PPM1A by K63-mediated linkage (Fig. 7b). Together, these data indicate that TRIM18 targets PPM1A and induces its ubiquitination for protein stability by K63-linkage.

PPM1A contains twenty-two lysine residues. Thirteen of these (Lys9, Lys12, Lys98, Lys118, Lys119, Lys156, Lys172, Lys208, Lys296, Lys303, Lys304, Lys310, and Lys354) were predicted to be possible ubiquitination sites with high scores by the BDM-PUB program (Additional file 1: Table S2). To determine the ubiquitination sites in PPM1A, we chose nine predicted ubiquitination sites with scores of more than one (Lys9, Lys12, Lys118, Lys119, Lys156, Lys296, Lys303, Lys304, and Lys354) and replaced each of those lysine residues individually with arginine. We co-expressed HA-tagged TRIM18 with Myc-tagged WT PPM1A and its mutants (K9RK12R, K118RK119R, K156R, K296R, K303RK304R, and K354R) in HEK293T cells and detected their expression and ubiquitination. TRIM18 was expressed similarly and could promote the stability of WT PPM1A and its mutants (K118RK119R, K156R, and K303RK304R), but not PPM1A mutants (K9RK12R, K296R and K354R) (Fig. 7c). In agreement, the total and K63-linked ubiquitination of PPM1A WT or its mutants (K118RK119R, K156R, and K303RK304R) was strongly enhanced by overexpression of TRIM18 (Fig. 7c). However, the total and K63-linked ubiquitination of PPM1A mutants (K9RK12R, K296R and K354R) by TRIM18 was absent (Fig. 7c). Furthermore, we examined the effect of PPM1A WT and its mutants on the IFN-β luciferase reporter activated by TBK1. Overexpression of PPM1A WT or its mutants (K118RK119R, K156R, and K303RK304R) reduced the IFN-β luciferase reporter activation by TBK1 (Fig. 7d). However, PPM1A mutants (K9RK12R, K296R and K354R) did not inhibit TBK1 mediated IFN-β luciferase reporter activation (Fig. 7d), indicating that the four lysine residues of PPM1A (Lys9, Lys12, Lys296, and Lys354) mediated its key effect on TBK1 triggered signaling activation. Next, we constructed PPM1A mutant harboring all those four lysine mutations (PPM1A K9/12/296/354R) to investigate if TRIM18 still induce ubiquitination of PPM1A mutant K9/12/296/354R. Indeed, TRIM18 could induce K63-linked ubiquitination of WT PPM1A, while the K63-mediated ubiquitination of PPM1A mutant K9/12/296/354R were completely blocked (Fig. 7e). The inhibition of PPM1A ubiquitination dramatically reduced its ability to dephosphorylate TBK1 and triggered much stronger phosphorylation and activation of TBK1 (Fig. 7e), thereby boosted TBK1 mediated IFN-β luciferase reporter activation by luciferase assay (Fig. 7f). Collectively, these data showed that TRIM18 targeted PPM1A and induced the K63-linked ubiquitination of PPM1A for maintaining its stability and four lysine residues (Lys9, Lys12, Lys296, and Lys354) were critical sites for TRIM18-mediated ubiquitination and regulation of PPM1A**.**

## Discussion

**Fig. 8:**
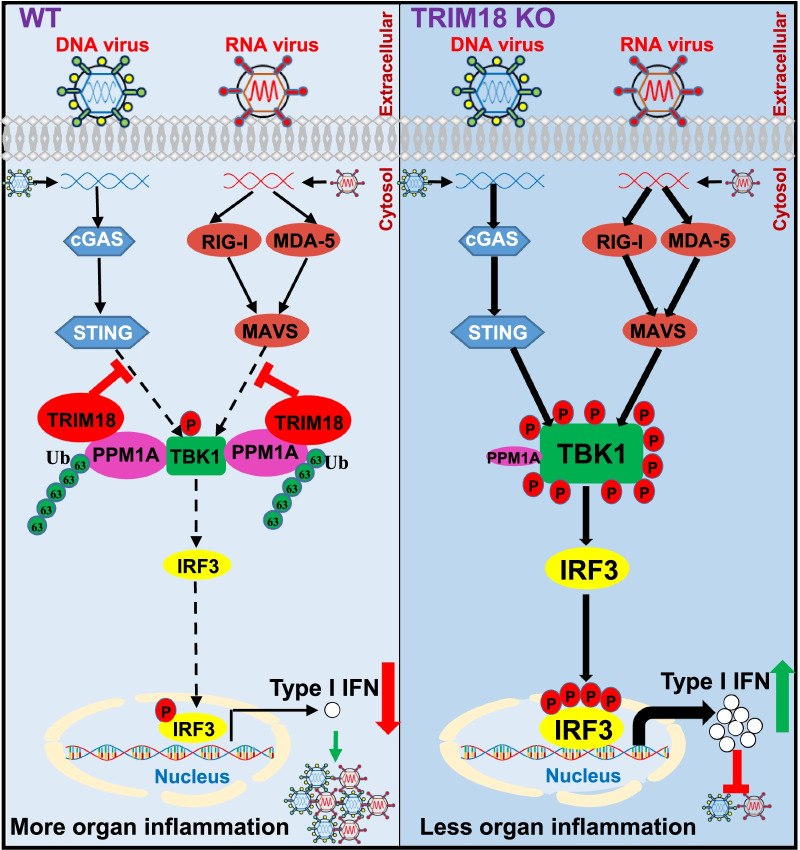
Schematic illustration of TRIM18 serving as a negative regulator in antiviral innate immunity against organ inflammations induced by RNA and DNA viruses. In WT macrophages after infections with DNA and RNA viruses, TRIM18 interacts with PPM1A and induces its K63-linked ubiquitination for maintaining stability of PPM1A, thereby further dephosphorylating TBK1 for its inactivation and blocking the interactions of TBK1 with its upstream adaptors STING and MAVS leading to dramatic reduction of type I IFN, which promotes viral myocarditis and more massive inflammations in lung and brain. In contrast, in TRIM18 KO macrophages post infections with DNA and RNA viruses, PPM1A could not maintain its stability without TRIM18, and loss its strong ability to inactivate TBK1 resulting in significant induction of type I IFN, which restricts viral myocarditis, inflammations in lung and brain

The elucidation of immune regulatory mechanisms is critical to understanding how the host constrains adventitious inflammation to maintain immune homeostasis. In the present study, we demonstrate an essential role of E3 ubiquitin ligase TRIM18 in serving as negative regulator of antiviral innate immunity against organ inflammations induced by RNA and DNA viruses (Fig. 8). TRIM18 was shown to reduce the type I IFN response by cytosolic dsRNA and dsDNA, which linked TRIM18 to both RNA and DNA sensing pathways. Deficiency of TRIM18 in both human and mouse macrophages potentiated type I IFN induction by RNA and DNA viruses. Functionally, knockout of TRIM18 protected mice from viral myocarditis, viral pneumonia, and herpes simplex encephalitis in vivo. Mechanistically, we demonstrated that TRIM18 recruited the protein phosphatase PPM1A to dephosphorylate TBK1, which inactivated TBK1 and blocked interactions of TBK1 with its upstream adaptors MAVS and STING, dampening type I IFN mediated antiviral signaling during virus infection. Furthermore, TRIM18 promoted K63-linked polyubiquitination of PPM1A for maintaining its stability. Importantly, ablation of TRIM18 led to enhanced antiviral cytokine production and clearance of both RNA and DNA viruses in vivo, underscoring its physiologic function. This work demonstrates that TRIM18 serves as an immunological rheostat to safeguard against inappropriate innate immune responses to cytosolic viral RNA and DNA in human and mouse macrophages. Since we only have TRIM18 global knockout mice but not macrophage specific TRIM18 knockout mice, we could not exclude the potential roles of TRIM18 in cells other than macrophages in antiviral innate immunity against RNA and DNA viruses. Because TRIM18 expression is induced by RNA and DNA virus infection and TRIM18 has high expression in lung, heart and brain of mice with HSV-1 infection, we speculate that the regulatory machinery of TRIM18 in antiviral innate immunity may exist in other cells types such as lung epithelial cells, cardiomyocytes or neurons.

Accumulating evidence suggests that members of the TRIM family play versatile roles in antiviral immunity [57, 58]. A previous systematic analysis of 75 human TRIM proteins indicated that nearly half of TRIM proteins serve as positive regulators of antiviral responses [58]. For example, TRIM4 and TRIM25 promote type I IFN production and NF-κB activity by regulating the ubiquitination of RIG-I in RNA sensing pathway [59, 60], while TRIM23-mediated K27-linked ubiquitination of NEMO promotes TLR3- and RIG-I/MDA5-mediated antiviral and inflammatory responses [61]. Additionally, TRIM32 and TRIM56 positively regulate DNA virus-induced type I IFN signaling by targeting STING for K63-linked ubiquitination [62, 63]. Furthermore, TRIM9 short isoform preferentially promotes DNA and RNA virus-induced production of type I interferon by recruiting GSK3β to TBK1 [64]. On contrast, TRIM38 is shown to negatively regulate TLR3/4-and RIG-I-mediated IFN-β production by promoting K48-linked polyubiquitination and proteasomal degradation of NAP1 [65]. However, these studies lack key in vivo data without using gene knockout mice. In our study, we demonstrated an essential role of TRIM18 in controlling viral myocarditis, viral pneumonia and herpes simplex encephalitis through downregulating innate immune activation against RNA and DNA viruses both in vitro and in vivo. We have previously demonstrated TRIM29 is a negative regulator in antiviral innate immunity against respiratory RNA virus by promoting K48-linked polyubiquitination and proteasomal degradation of NEMO in alveolar macrophages [38] and TRIM29 promotes DNA virus infection by targeting STING for K48-linked polyubiquitination and proteasomal degradation in airway epithelial cells and dendritic cells and targeting TAB2 for degradation in natural killer cells both in vitro and in vivo [39, 66]. Additionally, TRIM31 promotes activation of antiviral innate immunity against RNA viruses by inducing K63-linked polyubiquitination of MAVS using TRIM31 knockout mice [67]. Furthermore, TRIM21 could positively regulate IRF3-mediated type I IFN production through interaction with MAVS, thereby restricting RNA virus CVB3 replication and cardiac injury [68]. We compared the expression of TRIM21 and TRIM18 in CVB3 infected hearts and found TRIM18 expressed higher than TRIM21. We speculate that TRIM21 inhibits CVB3 induced myocarditis by serving as positive regulator in type I IFN production pathway, while TRIM18 promotes myocarditis induced by CVB3 through negatively regulating type I IFN production in antiviral innate immunity. Although TRIM29, TRIM31 and TRIM21 have essential roles in antiviral innate immunity in vivo, they only function their antiviral immunity against either RNA virus or DNA virus by targeting different substrates in different innate immune cells. Here, we identified TRIM18 as crucial negative regulator in antiviral immunity against both RNA and DNA viruses by recruiting PPM1A to inactivate TBK1 and block interactions of TBK1 with its upstream adaptors MAVS and STING for signal transduction in macrophages both in vitro and in vivo. It is reported that there are sex differences in immune responses that underlie COVID-19 disease outcomes [69] and men have higher COVID-19 mortality than women [70, 71]. Interestingly, TRIM18 gene is found on the X chromosome and mutations of TRIM18 gene are responsible for a rare genetic disease called X-linked Opitz G/BBB Syndrome (XLOS) [27]. Importantly, we found that male mice were more susceptible to CVB3 infection than females. Additionally, the public GEO profile database show that patients with SARS-CoV-2 infection have higher levels of TRIM18, while we demonstrated that WT mice with high expression of TRIM18 were more vulnerable to both RNA and DNA virus infections. Therefore, we hypothesize that TRIM18 may be a key biology factor associated with men’s higher risk of COVID-19-associated mortality, although testing this is beyond the scope of the current study.

TBK1 is a key adaptor protein shared by both RNA and DNA sensing pathways and is crucial for the activation of IRF3 and subsequent type I IFN induction [7, 72]. TBK1 is regulated by posttranslational modifications such as ubiquitination, phosphorylation, and acetylation. RNF128 promotes TBK1 activation by inducing its K63-linked polyubiquitination [73]. Likewise, TRIP, NLRP4-DTX4, Siglec1-TRIM27, and TRAF3IP3 also promote TBK1 degradation, although via K48-linked polyubiquitination [74–78]. It was reported that GSK3β, PPM1B, PP4 and PPM1A could modulate TBK1 activity by altering the TBK1 phosphorylation state [56, 79–81]. Additionally, HDAC9 deacetylates TBK1 and enhances TBK1 activation [82]. Although those above reports illustrate that TBK1 is under tight multi-layered control, the cellular regulatory mechanisms remain incompletely understood. Our data from IFN-β luciferase reporter and IP-MS assays suggest TRIM18 targets both RNA and DNA sensing pathways at TBK1 or its upstream nodes. In the macrophage steady state, TRIM18 interacts with PPM1A, which is reported to dephosphorylate and inactivate TBK1. Importantly, we detect interaction of TRIM18 with both PPM1A and TBK1 after virus infection, which drove us to further investigate the molecular mechanisms by which TRIM18 targets TBK1 to dampen type I IFN production in virus infection. Finally, we find TRIM18 serves as a negative regulator in antiviral innate immunity against RNA and DNA viruses through three novel mechanisms. First, TRIM18 recruits protein phosphatase PPM1A, which then interacts with and dephosphorylates TBK1 for its inactivation. Second, TRIM18 interacts with TBK1 and blocks its interactions with upstream adaptors MAVS and STING for preventing signal transduction during virus infection. Third, TRIM18 promotes the stability of PPM1A by inducing its K-63 linked polyubiquitination during virus infection. Furthermore, we found four lysine residues (Lys9, Lys12, Lys296, and Lys354) of PPM1A were critical sites for TRIM18-dependent ubiquitination and stability of PPM1A. Moreover, these four lysine residues of PPM1A mediated its inhibition of TBK1 triggered type I IFN production. Therefore, this work expands the regulatory landscape of the cytosolic sensing by RNA and DNA receptors and uncovers a function of TRIM18 in modulating innate immunity not previously appreciated.

## Conclusions

In summary, we have identified TRIM18 as a novel negative regulator of viral myocarditis, lung inflammation and brain damage by downregulating innate immune activation against both RNA and DNA viruses. Our results underscore the pivotal role of TRIM18 in controlling both RNA and DNA virus infections, where TRIM18 provides a safeguard against aberrant and excessive type I IFN production as well as potential tissue damage and autoimmunity diseases. Importantly, the ongoing COVID-19 pandemic serves as a reminder that the new emerging RNA viruses remain a significant public health threat [25]. Thus, our findings may provide an opportunity for boosting protective antiviral immunity by inhibiting TRIM18. Our work may also be beneficial in the design of better pharmacological antagonists to improve the vaccine efficacy against both RNA and DNA viruses including SARS-CoV-2.

## Supplementary Information

**Additional file 1: Table S1.** PPM1A is in the TRIM18-binding protein complex. **Table S2.** The potential ubiquitination sites at the PPM1A molecule. **Table S3.** Primers for qRT-PCR and PCR used in this study.

**Additional file 2: Fig S1.** TRIM18 inhibits production of type I IFN by human THP-1 macrophages after stimulation with dsRNA and dsDNA, but not LPS. **Fig S2. **TRIM18 negatively regulates IFN-α production in human THP-1 macrophages after infection with RNA and DNA viruses. **Fig S3.** *Trim18* gene targeting and TRIM18 expression in mouse macrophages and different tissues. **Fig S4.** TRIM18 does not affect expression of differentiation markers CD11b and F4/80 in mouse splenic macrophages. **Fig S5.** Knockout of TRIM18 enhances production of ISG15 and ISG56 in BMDM in response to dsRNA and dsDNA stimulations or infection with RNA and DNA viruses. **Fig S6.** TRIM18 is induced in human patients with SARS-CoV infection. **Fig S7.** TRIM18 inhibits IFN-β reporter activation mediated by overexpression of MDA5, MAVS, TBK1 and cGAS/STING, but not IKKi.

## Data Availability

All data relevant to the study are included in the article and in additional files. The reagents used in this publication are available from the corresponding author on reasonable request.
