# Supplementary material for: TRIM18 is a critical regulator of viral myocarditis and organ inflammation
Source: J Biomed Sci. 2022 Jul 31;29:55. doi: 10.1186/s12929-022-00840-z (PMC9339186; doi:10.1186/s12929-022-00840-z)
Supplement: Supplementary file 1 — Additional file 1: Table S1. PPM1A is in the TRIM18-binding protein complex. Table S2. The potential ubiquitination sites at the PPM1A molecule. Table S3. Primers for qRT-PCR and PCR used in this study. [file 12929_2022_840_MOESM1_ESM.docx]

**Additional file 1:** **Table S1. PPM1A is in the TRIM18-binding protein complex.**

| NCBI gi no. | Protein Name | Peptide Hits |
| --- | --- | --- |
| 255760028 | hematopoietic lineage cell-specific protein [Mus musculus] | 303 |
| 6671509 | actin, cytoplasmic 1 [Mus musculus] | 212 |
| 7106439 | tubulin beta-5 chain [Mus musculus] | 208 |
| 33859488 | tubulin beta-2A chain [Mus musculus] | 168 |
| 127139933 | E3 ubiquitin ligase TRIM18 (midline-1) [Mus musculus] | 163 |
| 19705578 | V-type proton ATPase subunit B, brain isoform [Mus musculus] | 137 |
| 254553321 | 40S ribosomal protein S3a [Mus musculus] | 124 |
| 31981382 | inosine-5'-monophosphate dehydrogenase 2 [Mus musculus] | 108 |
| 31981690 | heat shock cognate 71 kDa protein [Mus musculus] | 96 |
| 114326446 | myosin-9 isoform 1 [Mus musculus] | 87 |
| 6753832 | high affinity immunoglobulin gamma Fc receptor I precursor [Mus musculus] | 80 |
| 19527064 | V-type proton ATPase subunit B, kidney isoform [Mus musculus] | 72 |
| 40556608 | heat shock protein HSP 90-beta [Mus musculus] | 60 |
| 84662736 | 60S ribosomal protein L6 [Mus musculus] | 53 |
| 160333923 | heterogeneous nuclear ribonucleoprotein U [Mus musculus] | 48 |
| 124486698 | cyclin-dependent kinase 13 isoform 1 [Mus musculus] | 41 |
| 6679443 | protein phosphatase 1A (PPM1A) [Mus musculus] | 38 |
| 20982845 | RNA-binding protein FUS [Mus musculus] | 35 |
| 257196186 | poly(U)-binding-splicing factor PUF60 isoform c [Mus musculus] | 31 |
| 18959280 | serine/threonine-protein kinase Sgk3 [Mus musculus] | 27 |
| 33859482 | elongation factor 2 [Mus musculus] | 20 |
| 228480232 | U2 small nuclear ribonucleoprotein A' [Mus musculus] | 18 |
| 112293273 | DNA replication licensing factor MCM5 [Mus musculus] | 16 |
| 15426055 | coatomer subunit beta [Mus musculus] | 15 |
| 145207948 | E3 ubiquitin/ISG15 ligase TRIM25 [Mus musculus] | 14 |
| 112734861 | importin-9 [Mus musculus] | 13 |
| 6679443 | protein phosphatase 1A [Mus musculus] | 12 |
| 119226245 | serine/arginine-rich splicing factor 5 [Mus musculus] | 12 |
| 93102413 | galactokinase [Mus musculus] | 11 |
| 38604071 | exportin-1 [Mus musculus] | 10 |
| 13386106 | cleavage and polyadenylation specificity factor subunit 5 [Mus musculus] | 9 |
| 6996911 | argininosuccinate synthase [Mus musculus] | 8 |
| 14149756 | eukaryotic translation initiation factor 2 subunit 2 [Mus musculus] | 7 |
| 170014720 | D-beta-hydroxybutyrate dehydrogenase, mitochondrial precursor [Mus musculus] | 6 |
| 113199765 | cell cycle checkpoint protein RAD17 [Mus musculus] | 5 |
| 10946722 | cell differentiation protein RCD1 homolog [Mus musculus] | 5 |
| 71534295 | endoplasmic reticulum mannosyl-oligosaccharide 1,2-alpha-mannosidase [Mus musculus] | 4 |
| 145966883 | 26S proteasome non-ATPase regulatory subunit 14 [Mus musculus] | 3 |
| 31543940 | vesicle-associated membrane protein-associated protein B [Mus musculus] | 3 |
| 304555605 | immunoglobulin-binding protein 1 [Mus musculus] | 2 |
| 160333109 | B-cell differentiation antigen CD72 isoform 4 [Mus musculus] | 2 |
| 21704220 | charged multivesicular body protein 1a [Mus musculus] | 2 |
| 19527092 | regulator of chromosome condensation isoform 2 [Mus musculus] | 2 |
| 42476274 | xaa-Pro aminopeptidase 1 [Mus musculus] | 2 |
| 118150670 | zinc transporter SLC39A7 precursor [Mus musculus] | 2 |
| 254675217 | vacuolar protein sorting-associated protein 18 homolog [Mus musculus] | 2 |
| 19527242 | tubulin gamma-1 chain [Mus musculus] | 2 |

Mouse BMDM lysate was prepared, followed by anti-TRIM18 immunoprecipitation and protein sequencing by liquid chromatography-mass spectrometry. NCBI gi no: unique protein identification number; Hits: the number of peptides ions matched that associated protein.

**Additional file 1:** **Table S2. The potential ubiquitination sites at the PPM1A molecule.**

Mouse PPM1A Ubiquitination sites:

MGAFLDKPKMEKHNAQGQGNGLRYGLSSMQGWRVEMEDAHTAVIGLPSGLETWSFFAVYDGHAGSQVAKYCCEHLLDHITNNQDFRGSAGAPSVENVKNGIRTGFLEIDEHMRVMSEKKHGADRSGSTAVGVLISPQHTYFINCGDSRGLLCRNRKVHFFTQDHKPSNPLEKERIQNAGGSVMIQRVNGSLAVSRALGDFDYKCVHGKGPTEQLVSPEPEVHDIERSEEDDQFIILACDGIWDVMGNEELCDFVRSRLEVTDDLEKVCNEVVDTCLYKGSRDNMSVILICFPSAPKVSAEAVKKEAELDKYLESRVEEIIKKQVEGVPDLVHVMRTLASENIPSLPPGGELASKRNVIEAVYNRLNPYKNDDTDSASTDDMW

| **Peptide** | **Position** | **Score** | **Threshold** |
| --- | --- | --- | --- |
| GAFLDKPKMEKHNAQ | 9 | 1.27 | 0.3 |
| LDKPKMEKHNAQGQG | 12 | 1.44 | 0.3 |
| APSVENVKNGIRTGF | 98 | 0.39 | 0.3 |
| HMRVMSEKKHGADRS | 118 | 0.94 | 0.3 |
| MRVMSEKKHGADRSG | 119 | 1.28 | 0.3 |
| GLLCRNRKVHFFTQD | 156 | 1.10 | 0.3 |
| KPSNPLEKERIQNAG | 172 | 0.87 | 0.3 |
| DYKCVHGKGPTEQLV | 208 | 0.58 | 0.3 |
| ICFPSAPKVSAEAVK | 296 | 1.88 | 0.3 |
| KVSAEAVKKEAELDK | 303 | 1.84 | 0.3 |
| VSAEAVKKEAELDKY | 304 | 1.16 | 0.3 |
| KKEAELDKYLESRVE | 310 | 0.69 | 0.3 |
| PGGELASKRNVIEAV | 354 | 2.70 | 0.3 |

The possibility of ubiquitination of lysine residues in PPM1A (bottom panel) was predicted from the amino acid sequence of PPM1A (top panel) by “BDM-PUB: Prediction of Ubiquitination sites with Bayesian Discriminant Methods”.

**Additional file 1:** **Table S3**. **Primers for qRT-PCR and PCR used in this study.**

| Gene | Sequence |
| --- | --- |
| qRT-PCR |  |
| Mouse *BNP* | F: 5’- AAGTCCTAGCCAGTCTCCAGA -3’ |
|  | R: 5’- GAGCTGTCTCTGGGCCATTTC -3’ |
| Mouse *Gapdh* | F: 5’- AGGTCGGTGTGAACGGATTTG -3’ |
|  | R: 5’- TGTAGACCATGTAGTTGAGGTCA -3’ |
| Mouse *β-actin* | F: 5’- CGTGAAAAGATGACCCAGATCA -3’ |
|  | R: 5’- CACAGCCTGGATGGCTACGT -3’ |
| Mouse *ISG15* | F: 5’- CATCCTGGTGAGGAACGAAAGG -3’ |
|  | R: 5’- CTCAGCCAGAACTGGTCTTCGT -3’ |
| Mouse *ISG56* | F: 5’- TACAGGCTGGAGTGTGCTGAGA -3’ |
|  | R: 5’- CTCCACTTTCAGAGCCTTCGCA -3’ |
| Mouse *Trim18* | F: 5’- TGAATATGTACTGTGTGACCGATGA -3’ |
|  | R: 5’- TGGTGAGATTACTCTCCAAGTTTTG -3’ |
| Trim18 genotype PCR |  |
| LacZ F | 5’-TCCCAACAGTTGCGCAGCCTGAATG -3’ |
| LacZ R | 5’-ATATCCTGATCTTCCAGATAACTGCCG -3’ |
| Exon 2 F | 5’-TGGAGTCGGAGCTGACCTGTCCTATTTG -3’ |
| Exon 2 R | 5’-CAGAGGACATGGTGTTGGCGTCAAAGGC -3’ |
