# Supplementary material for: TRIM18 is a critical regulator of viral myocarditis and organ inflammation
Source: J Biomed Sci. 2022 Jul 31;29:55. doi: 10.1186/s12929-022-00840-z (PMC9339186; doi:10.1186/s12929-022-00840-z)
Supplement: Supplementary file 2 — Additional file 2: Fig S1. TRIM18 inhibits production of type I IFN by human THP-1 macrophages after stimulation with dsRNA and dsDNA, but not LPS. Fig S2. TRIM18 negatively regulates IFN-α production in human THP-1 macrophages after infection with RNA and DNA viruses. Fig S3. Trim18 gene targeting and TRIM18 expression in mouse macrophages and different tissues. Fig S4. TRIM18 does not affect expression of differentiation markers CD11b and F4/80 in mouse splenic macrophages. Fig S5. Knockout of TRIM18 enhances production of ISG15 and ISG56 in BMDM in response to dsRNA and dsDNA stimulations or infection with RNA and DNA viruses. Fig S6. TRIM18 is induced in human patients with SARS-CoV infection. Fig S7. TRIM18 inhibits IFN-β reporter activation mediated by overexpression of MDA5, MAVS, TBK1 and cGAS/STING, but not IKKi. [file 12929_2022_840_MOESM2_ESM.pptx]

## Slide 1
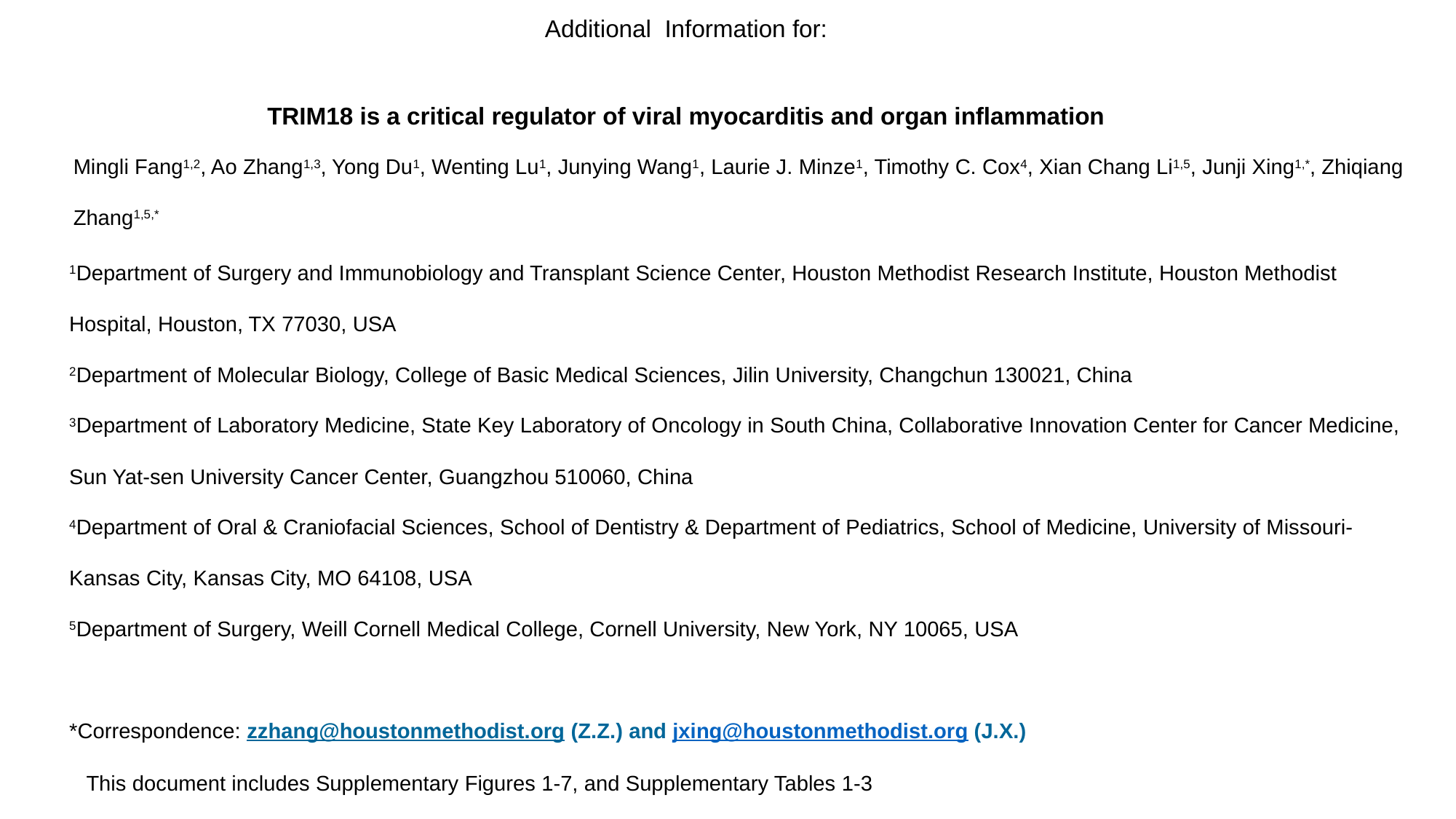

Additional Information for:
TRIM18 is a critical regulator of viral myocarditis and organ inflammation
Mingli Fang1,2, Ao Zhang1,3, Yong Du1, Wenting Lu1, Junying Wang1, Laurie J. Minze1, Timothy C. Cox4, Xian Chang Li1,5, Junji Xing1,*, Zhiqiang Zhang1,5,*
1Department of Surgery and Immunobiology and Transplant Science Center, Houston Methodist Research Institute, Houston Methodist Hospital, Houston, TX 77030, USA
2Department of Molecular Biology, College of Basic Medical Sciences, Jilin University, Changchun 130021, China
3Department of Laboratory Medicine, State Key Laboratory of Oncology in South China, Collaborative Innovation Center for Cancer Medicine, Sun Yat-sen University Cancer Center, Guangzhou 510060, China
4Department of Oral & Craniofacial Sciences, School of Dentistry & Department of Pediatrics, School of Medicine, University of Missouri-Kansas City, Kansas City, MO 64108, USA
5Department of Surgery, Weill Cornell Medical College, Cornell University, New York, NY 10065, USA
*Correspondence: zzhang@houstonmethodist.org (Z.Z.) and jxing@houstonmethodist.org (J.X.)
This document includes Supplementary Figures 1-7, and Supplementary Tables 1-3

## Slide 2
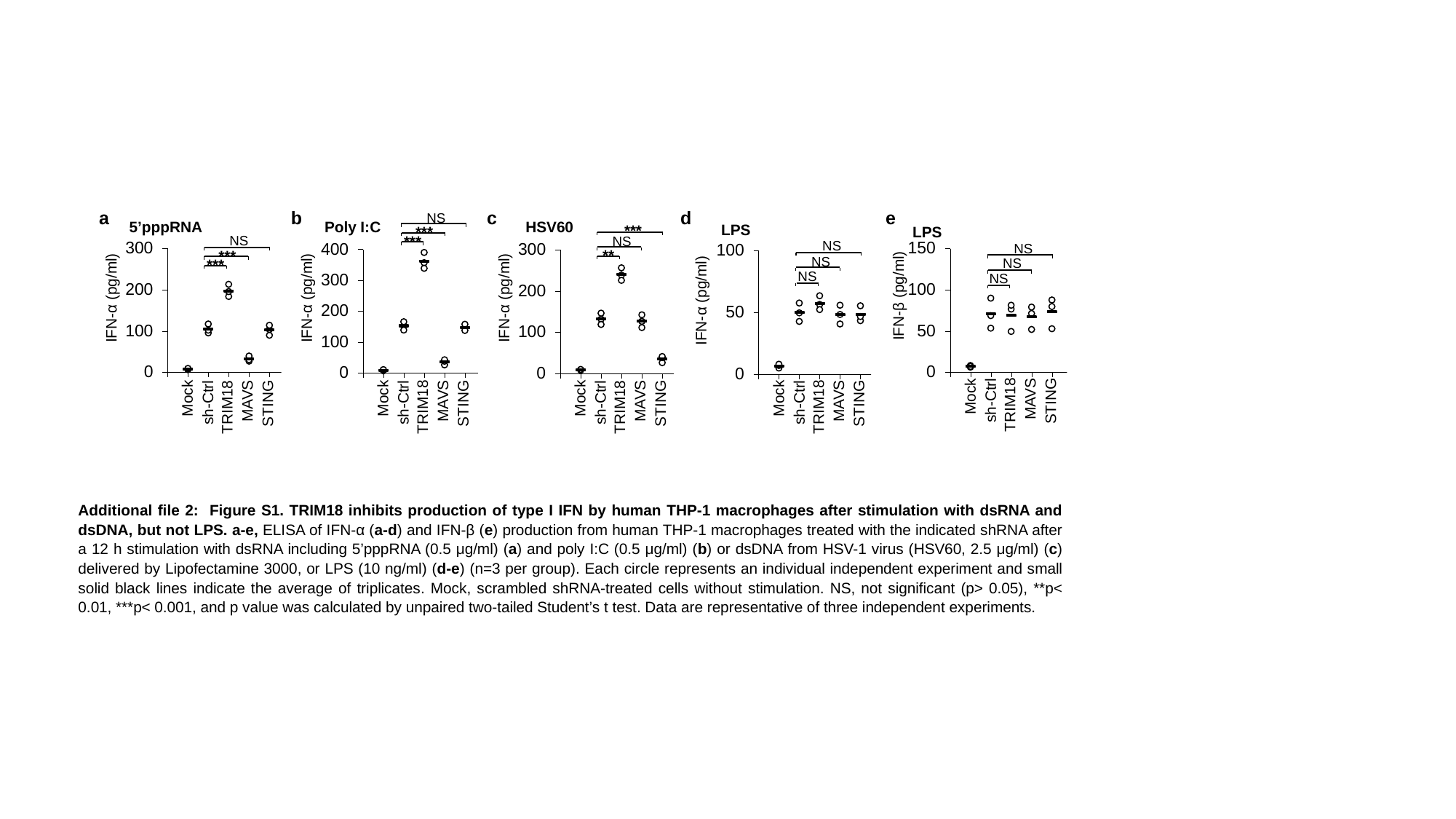

a
b
c
d
e
 NS
 ***
 ***
5’pppRNA
Poly I:C
HSV60
LPS
LPS
 ***
 NS
 **
 NS
 ***
 ***
 NS
 NS
 NS
 NS
 NS
 NS
IFN-β (pg/ml)
IFN-α (pg/ml)
IFN-α (pg/ml)
IFN-α (pg/ml)
IFN-α (pg/ml)
Mock
sh-Ctrl
MAVS
STING
TRIM18
Mock
sh-Ctrl
MAVS
STING
TRIM18
Mock
sh-Ctrl
MAVS
STING
TRIM18
Mock
sh-Ctrl
MAVS
STING
TRIM18
Mock
sh-Ctrl
MAVS
STING
TRIM18
Additional file 2: Figure S1. TRIM18 inhibits production of type I IFN by human THP-1 macrophages after stimulation with dsRNA and dsDNA, but not LPS. a-e, ELISA of IFN-α (a-d) and IFN-β (e) production from human THP-1 macrophages treated with the indicated shRNA after a 12 h stimulation with dsRNA including 5’pppRNA (0.5 μg/ml) (a) and poly I:C (0.5 μg/ml) (b) or dsDNA from HSV-1 virus (HSV60, 2.5 μg/ml) (c) delivered by Lipofectamine 3000, or LPS (10 ng/ml) (d-e) (n=3 per group). Each circle represents an individual independent experiment and small solid black lines indicate the average of triplicates. Mock, scrambled shRNA-treated cells without stimulation. NS, not significant (p> 0.05), **p< 0.01, ***p< 0.001, and p value was calculated by unpaired two-tailed Student’s t test. Data are representative of three independent experiments.

## Slide 3
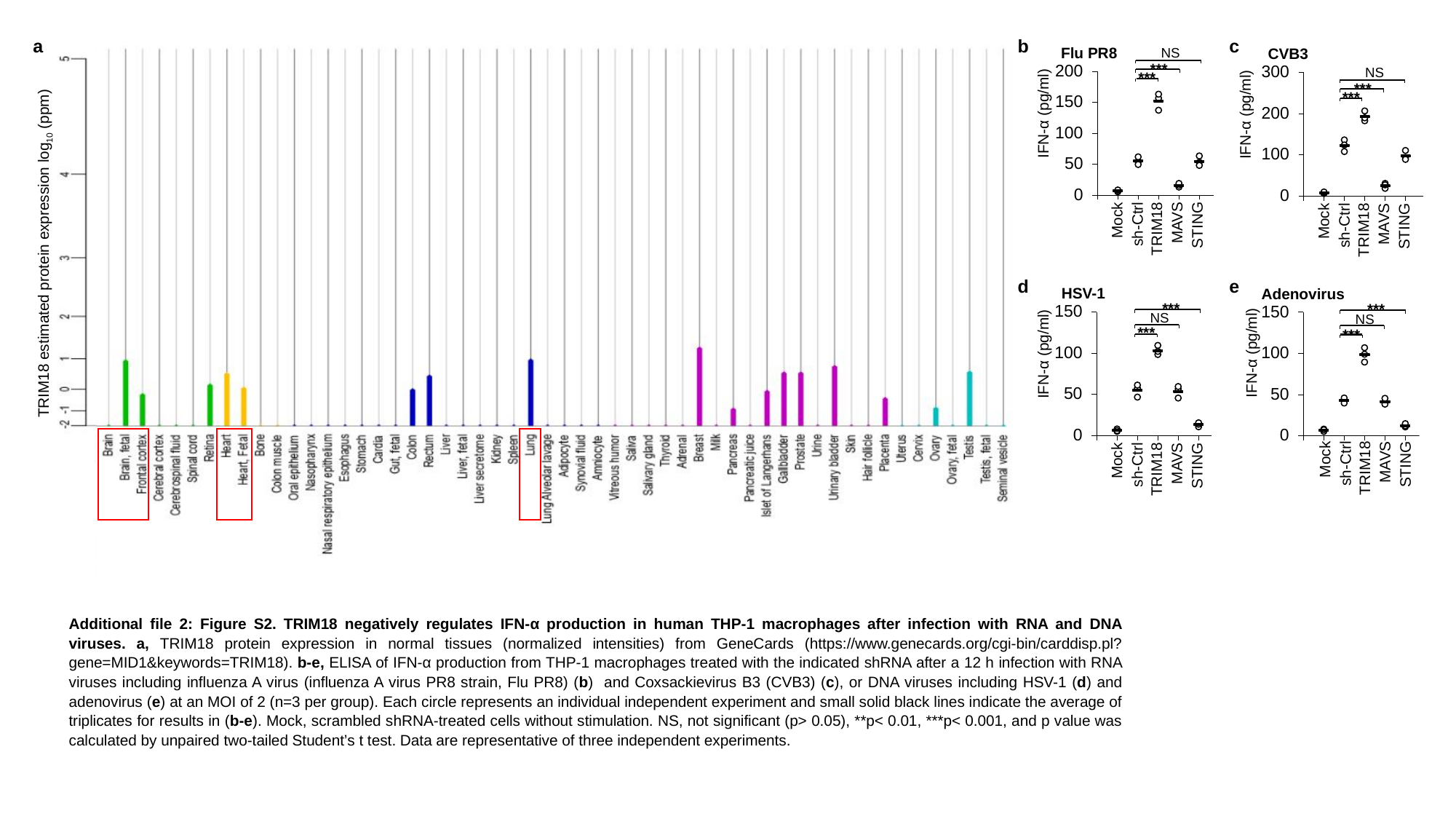

a
b
c
Flu PR8
CVB3
TRIM18 estimated protein expression log10 (ppm)
 NS
 ***
 ***
 NS
 ***
 ***
IFN-α (pg/ml)
IFN-α (pg/ml)
Mock
sh-Ctrl
MAVS
STING
TRIM18
Mock
sh-Ctrl
MAVS
STING
TRIM18
d
e
HSV-1
Adenovirus
 ***
 NS
 ***
 ***
 NS
 ***
IFN-α (pg/ml)
IFN-α (pg/ml)
Mock
sh-Ctrl
MAVS
STING
TRIM18
Mock
sh-Ctrl
MAVS
STING
TRIM18
Additional file 2: Figure S2. TRIM18 negatively regulates IFN-α production in human THP-1 macrophages after infection with RNA and DNA viruses. a, TRIM18 protein expression in normal tissues (normalized intensities) from GeneCards (https://www.genecards.org/cgi-bin/carddisp.pl?gene=MID1&keywords=TRIM18). b-e, ELISA of IFN-α production from THP-1 macrophages treated with the indicated shRNA after a 12 h infection with RNA viruses including influenza A virus (influenza A virus PR8 strain, Flu PR8) (b) and Coxsackievirus B3 (CVB3) (c), or DNA viruses including HSV-1 (d) and adenovirus (e) at an MOI of 2 (n=3 per group). Each circle represents an individual independent experiment and small solid black lines indicate the average of triplicates for results in (b-e). Mock, scrambled shRNA-treated cells without stimulation. NS, not significant (p> 0.05), **p< 0.01, ***p< 0.001, and p value was calculated by unpaired two-tailed Student’s t test. Data are representative of three independent experiments.

## Slide 4
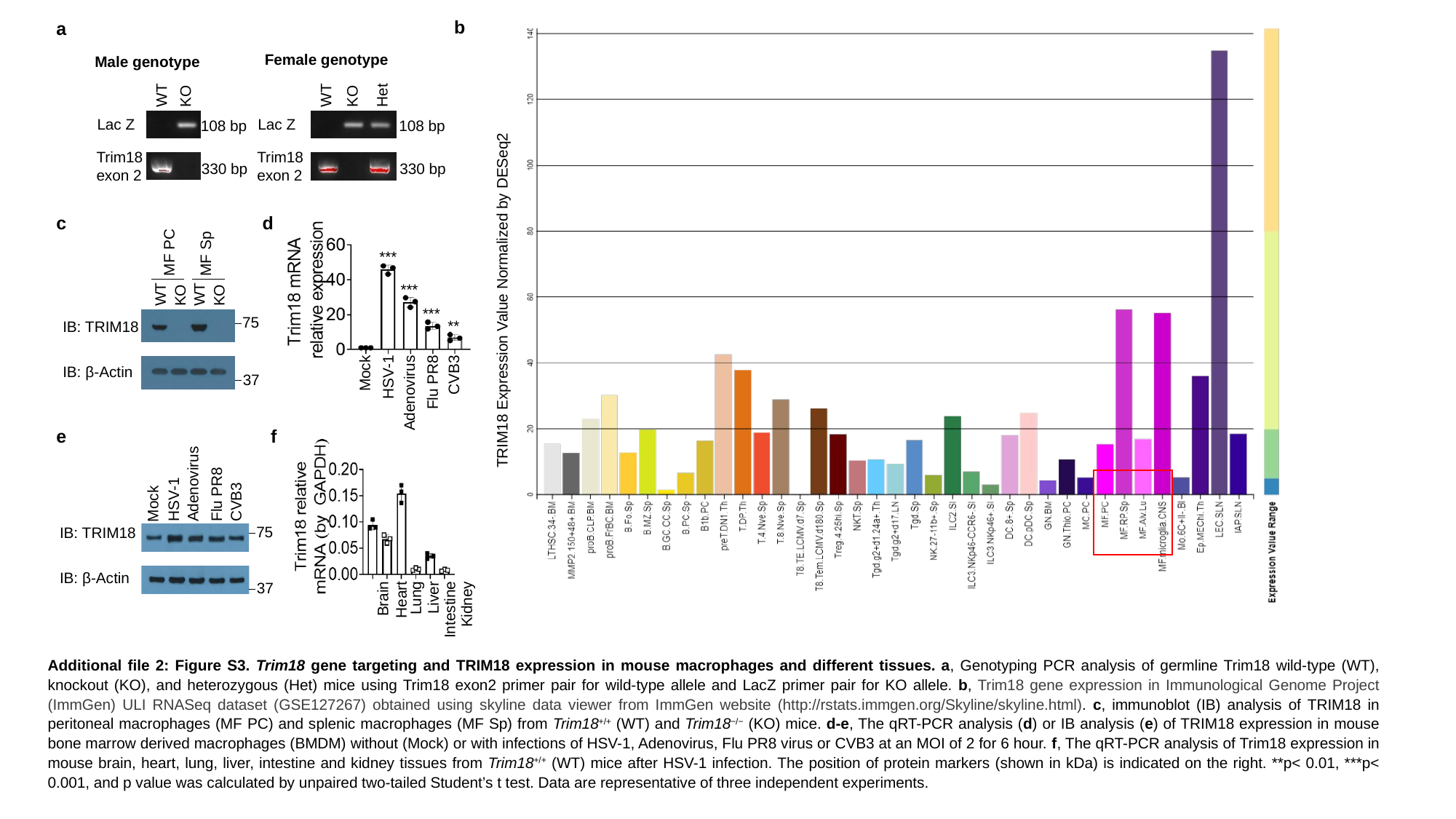

b
a
Female genotype
Male genotype
Het
WT
WT
KO
KO
Lac Z
Lac Z
108 bp
108 bp
Trim18
exon 2
Trim18
exon 2
330 bp
330 bp
c
d
***
***
***
**
Mock
HSV-1
Flu PR8
CVB3
Adenovirus
MF PC
MF Sp
WT
WT
KO
KO
75
IB: TRIM18
IB: β-Actin
37
TRIM18 Expression Value Normalized by DESeq2
e
f
Brain
Heart
Lung
Liver
Kidney
Intestine
Adenovirus
Flu PR8
HSV-1
CVB3
Mock
75
IB: TRIM18
IB: β-Actin
37
Additional file 2: Figure S3. Trim18 gene targeting and TRIM18 expression in mouse macrophages and different tissues. a, Genotyping PCR analysis of germline Trim18 wild-type (WT), knockout (KO), and heterozygous (Het) mice using Trim18 exon2 primer pair for wild-type allele and LacZ primer pair for KO allele. b, Trim18 gene expression in Immunological Genome Project (ImmGen) ULI RNASeq dataset (GSE127267) obtained using skyline data viewer from ImmGen website (http://rstats.immgen.org/Skyline/skyline.html). c, immunoblot (IB) analysis of TRIM18 in peritoneal macrophages (MF PC) and splenic macrophages (MF Sp) from Trim18+/+ (WT) and Trim18−/− (KO) mice. d-e, The qRT-PCR analysis (d) or IB analysis (e) of TRIM18 expression in mouse bone marrow derived macrophages (BMDM) without (Mock) or with infections of HSV-1, Adenovirus, Flu PR8 virus or CVB3 at an MOI of 2 for 6 hour. f, The qRT-PCR analysis of Trim18 expression in mouse brain, heart, lung, liver, intestine and kidney tissues from Trim18+/+ (WT) mice after HSV-1 infection. The position of protein markers (shown in kDa) is indicated on the right. **p< 0.01, ***p< 0.001, and p value was calculated by unpaired two-tailed Student’s t test. Data are representative of three independent experiments.

## Slide 5
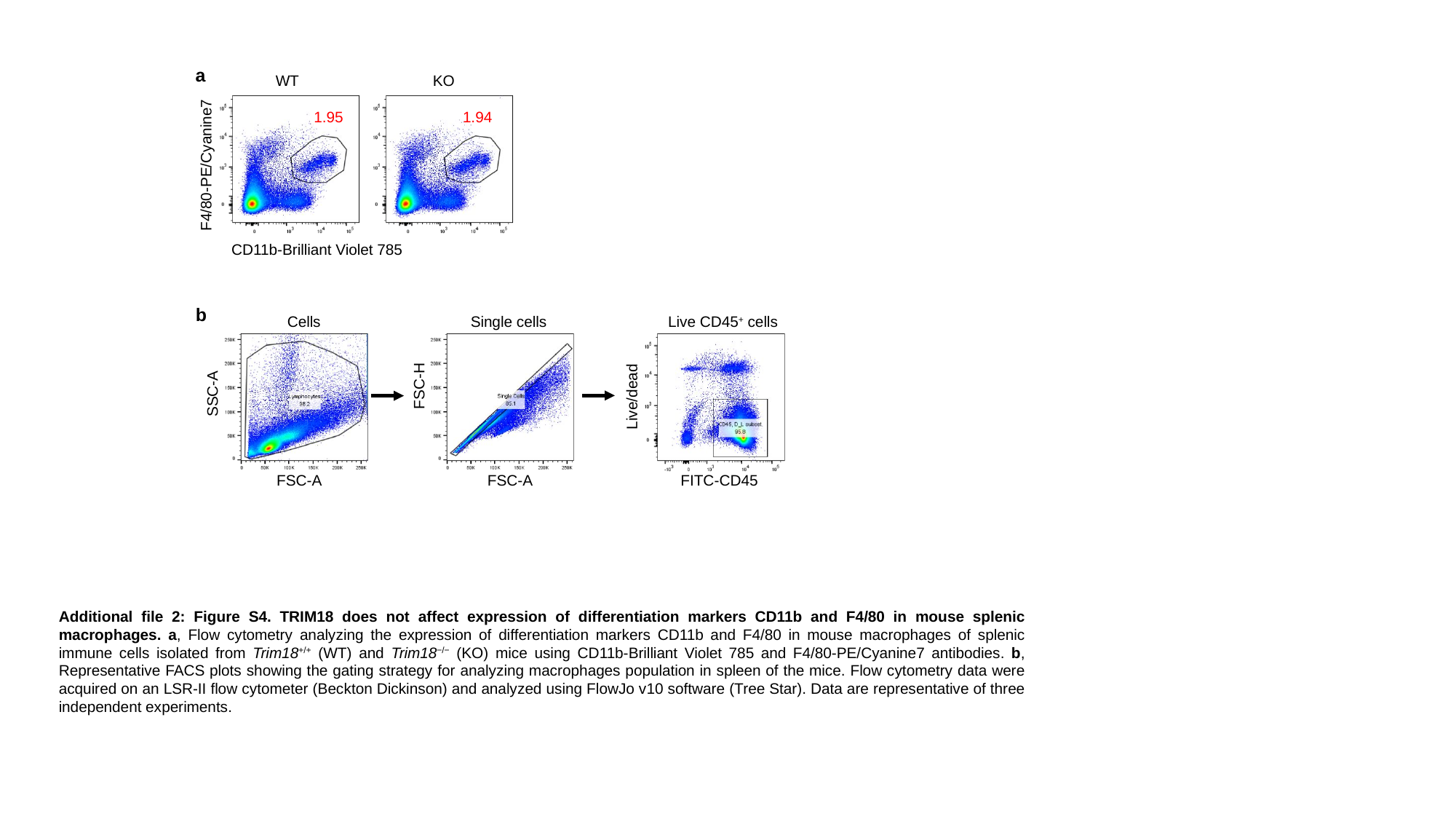

a
WT
KO
1.95
1.94
F4/80-PE/Cyanine7
CD11b-Brilliant Violet 785
b
Cells
Single cells
Live CD45+ cells
FSC-H
SSC-A
Live/dead
FSC-A
FSC-A
FITC-CD45
Additional file 2: Figure S4. TRIM18 does not affect expression of differentiation markers CD11b and F4/80 in mouse splenic macrophages. a, Flow cytometry analyzing the expression of differentiation markers CD11b and F4/80 in mouse macrophages of splenic immune cells isolated from Trim18+/+ (WT) and Trim18−/− (KO) mice using CD11b-Brilliant Violet 785 and F4/80-PE/Cyanine7 antibodies. b, Representative FACS plots showing the gating strategy for analyzing macrophages population in spleen of the mice. Flow cytometry data were acquired on an LSR-II flow cytometer (Beckton Dickinson) and analyzed using FlowJo v10 software (Tree Star). Data are representative of three independent experiments.

## Slide 6
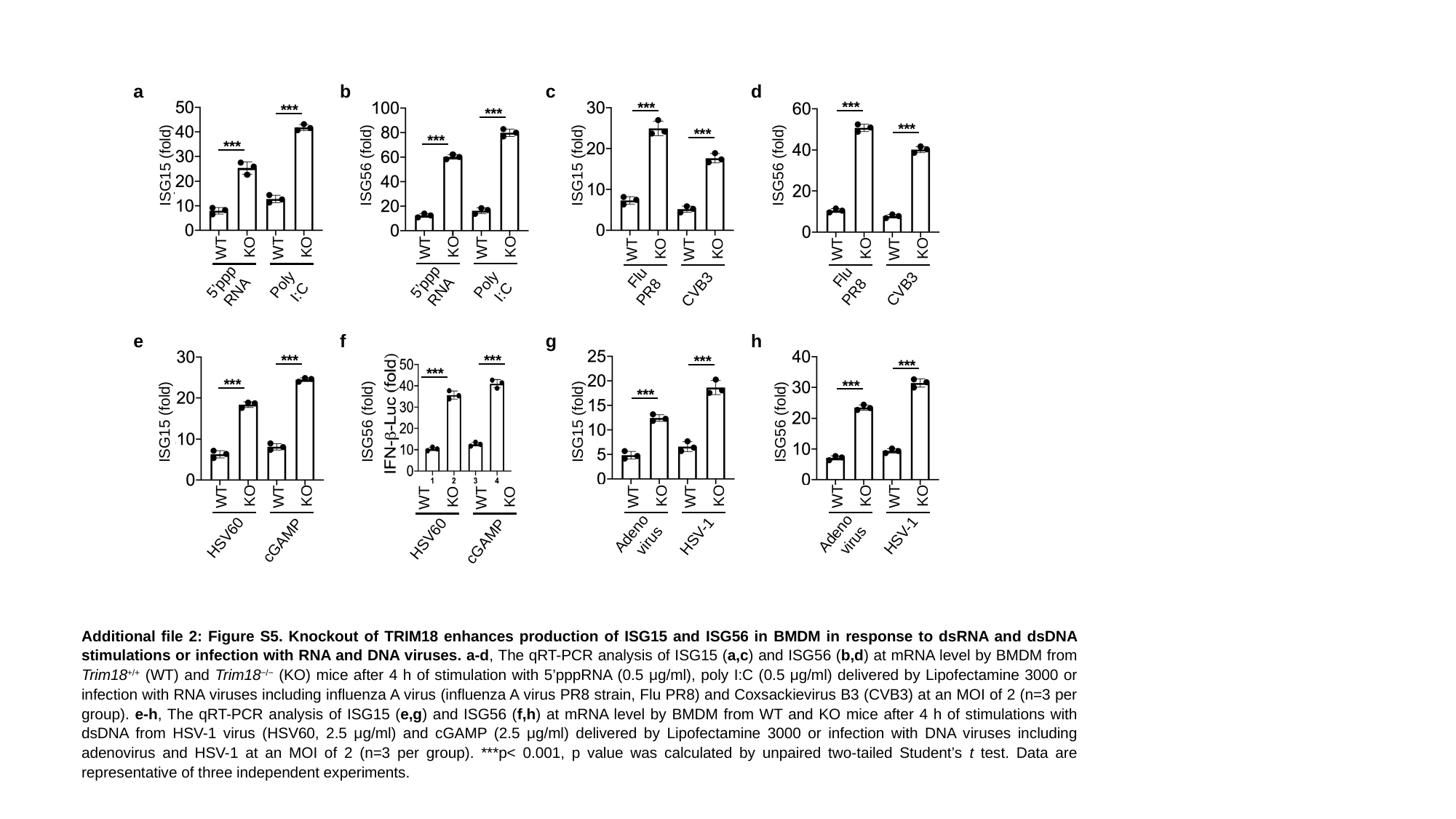

a
b
c
d
 ***
 ***
 ***
 ***
 ***
 ***
 ***
 ***
ISG15 (fold)
ISG56 (fold)
ISG15 (fold)
ISG56 (fold)
WT
KO
WT
KO
5’ppp
RNA
Poly I:C
WT
KO
WT
KO
5’ppp
RNA
Poly I:C
WT
KO
WT
KO
Flu
PR8
CVB3
WT
KO
WT
KO
Flu
PR8
CVB3
e
f
g
h
 ***
 ***
 ***
 ***
 ***
 ***
 ***
 ***
ISG15 (fold)
ISG56 (fold)
ISG15 (fold)
ISG56 (fold)
WT
KO
WT
KO
Adeno
virus
HSV-1
WT
KO
WT
KO
Adeno
virus
HSV-1
WT
KO
WT
KO
HSV60
cGAMP
WT
KO
WT
KO
HSV60
cGAMP
Additional file 2: Figure S5. Knockout of TRIM18 enhances production of ISG15 and ISG56 in BMDM in response to dsRNA and dsDNA stimulations or infection with RNA and DNA viruses. a-d, The qRT-PCR analysis of ISG15 (a,c) and ISG56 (b,d) at mRNA level by BMDM from Trim18+/+ (WT) and Trim18−/− (KO) mice after 4 h of stimulation with 5’pppRNA (0.5 μg/ml), poly I:C (0.5 μg/ml) delivered by Lipofectamine 3000 or infection with RNA viruses including influenza A virus (influenza A virus PR8 strain, Flu PR8) and Coxsackievirus B3 (CVB3) at an MOI of 2 (n=3 per group). e-h, The qRT-PCR analysis of ISG15 (e,g) and ISG56 (f,h) at mRNA level by BMDM from WT and KO mice after 4 h of stimulations with dsDNA from HSV-1 virus (HSV60, 2.5 μg/ml) and cGAMP (2.5 μg/ml) delivered by Lipofectamine 3000 or infection with DNA viruses including adenovirus and HSV-1 at an MOI of 2 (n=3 per group). ***p< 0.001, p value was calculated by unpaired two-tailed Student’s t test. Data are representative of three independent experiments.

## Slide 7
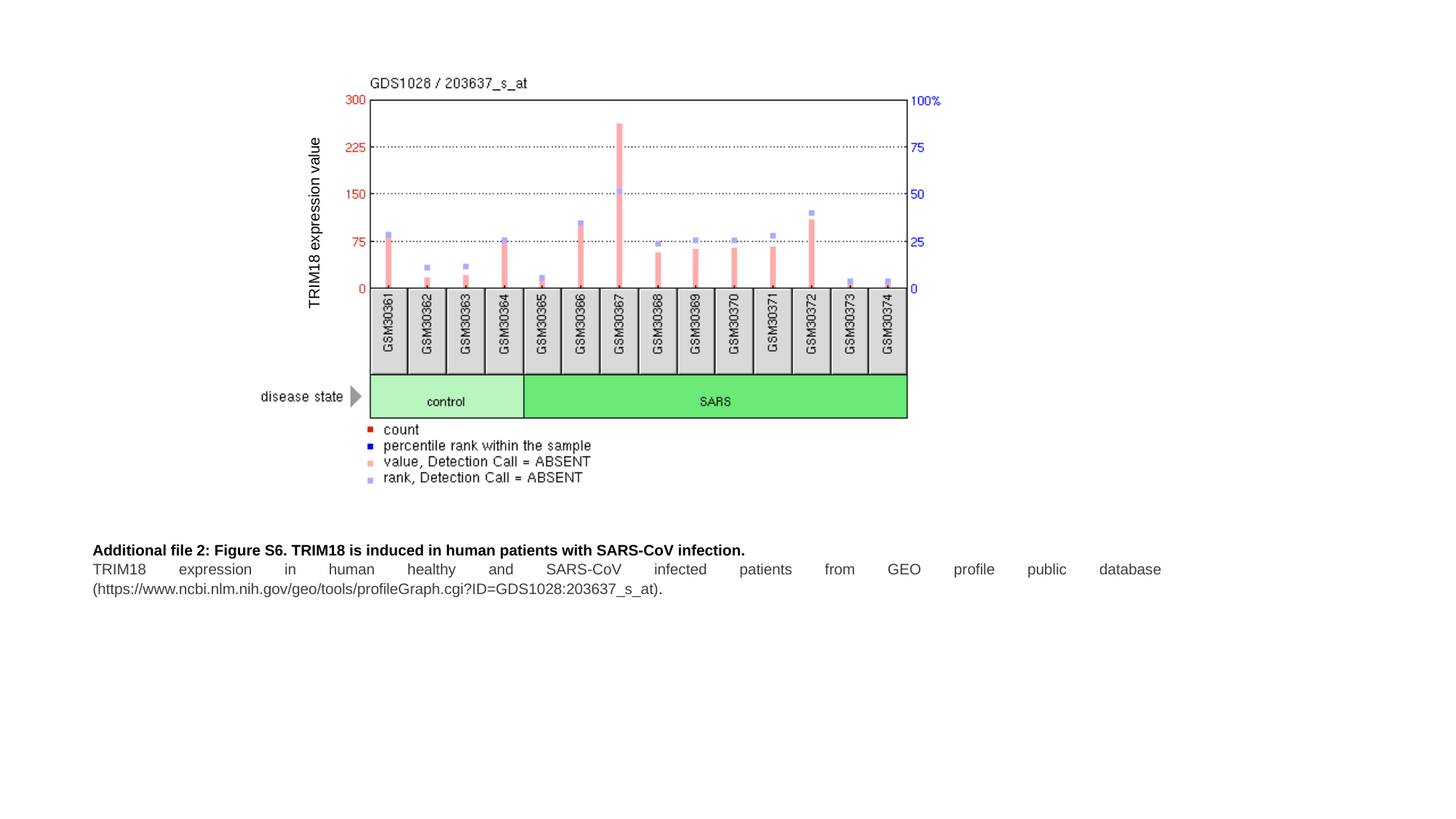

TRIM18 expression value
Additional file 2: Figure S6. TRIM18 is induced in human patients with SARS-CoV infection.
TRIM18 expression in human healthy and SARS-CoV infected patients from GEO profile public database (https://www.ncbi.nlm.nih.gov/geo/tools/profileGraph.cgi?ID=GDS1028:203637_s_at).

## Slide 8
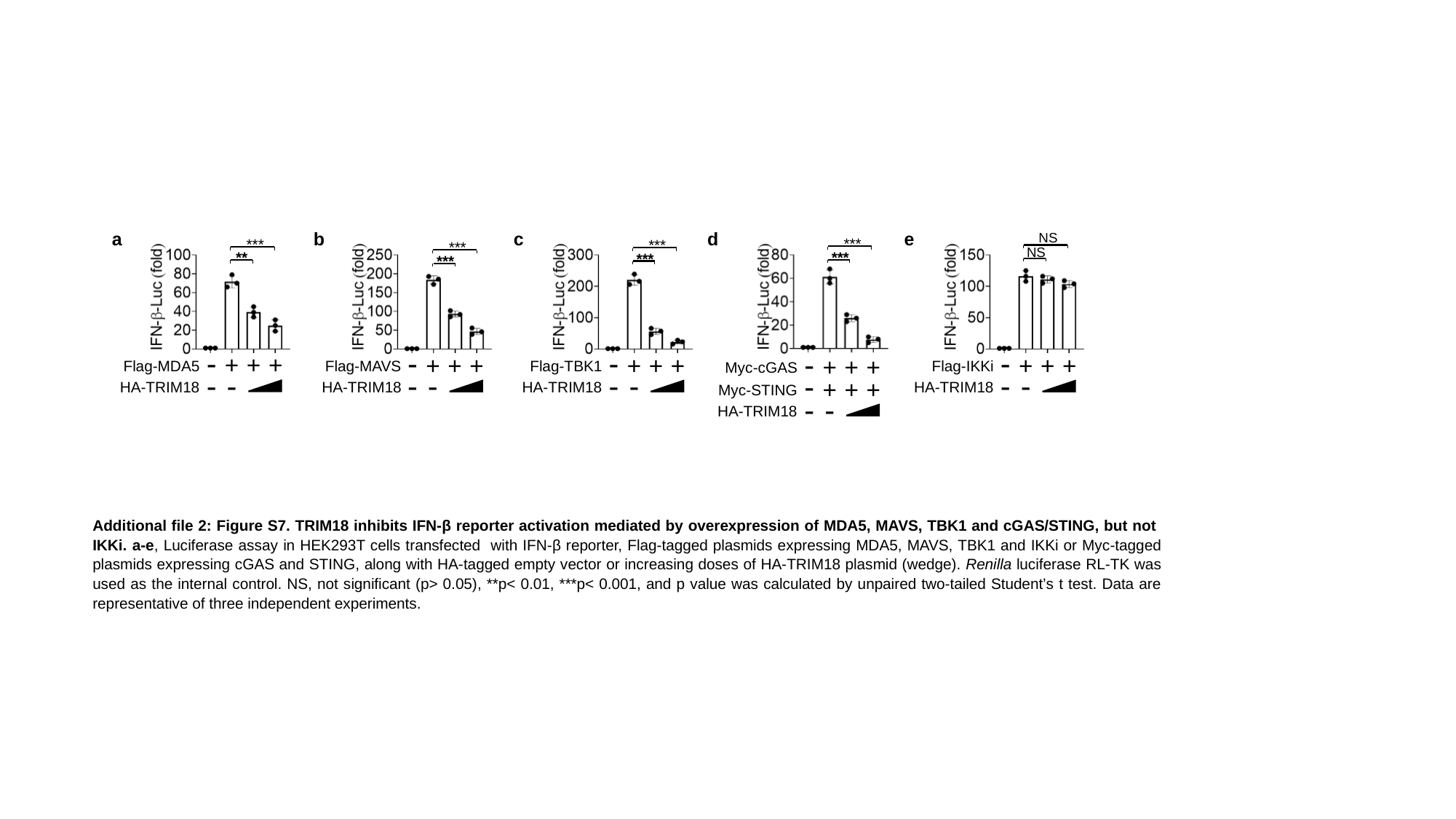

a
b
c
d
e
 NS
 NS
 ***
 ***
 ***
 **
 ***
 ***
 ***
 ***
-
+
+
+
Flag-MDA5
-
-
HA-TRIM18
-
+
+
+
Flag-IKKi
-
-
HA-TRIM18
-
+
+
+
Flag-MAVS
-
-
HA-TRIM18
-
+
+
+
Flag-TBK1
-
-
HA-TRIM18
-
+
+
+
Myc-cGAS
-
+
+
+
Myc-STING
-
-
HA-TRIM18
Additional file 2: Figure S7. TRIM18 inhibits IFN-β reporter activation mediated by overexpression of MDA5, MAVS, TBK1 and cGAS/STING, but not IKKi. a-e, Luciferase assay in HEK293T cells transfected with IFN-β reporter, Flag-tagged plasmids expressing MDA5, MAVS, TBK1 and IKKi or Myc-tagged plasmids expressing cGAS and STING, along with HA-tagged empty vector or increasing doses of HA-TRIM18 plasmid (wedge). Renilla luciferase RL-TK was used as the internal control. NS, not significant (p> 0.05), **p< 0.01, ***p< 0.001, and p value was calculated by unpaired two-tailed Student’s t test. Data are representative of three independent experiments.
